# Supplementary material for: Systematic Assessment of Transcriptomic Biomarkers for Immune Checkpoint Blockade Response in Cancer Immunotherapy
Source: Cancers (Basel). 2021 Apr 1;13(7):1639. doi: 10.3390/cancers13071639 (PMC8037221; doi:10.3390/cancers13071639)
Supplement: Supplementary file 1 [file cancers-13-01639-s001.zip › cancers-1107663-Supplementary File.docx]

Article

Supplemental Materials: Systematic Assessment of Transcriptomic Biomarkers for Immune Checkpoint Blockade Response in Cancer Immunotherapy

Shangqin Sun, Liwen Xu, Xinxin Zhang, Lin Pang, Zhilin Long, Chunyu Deng, Jiali Zhu, Shuting Zhou, Linyun Wan, Bo Pang and Yun Xiao


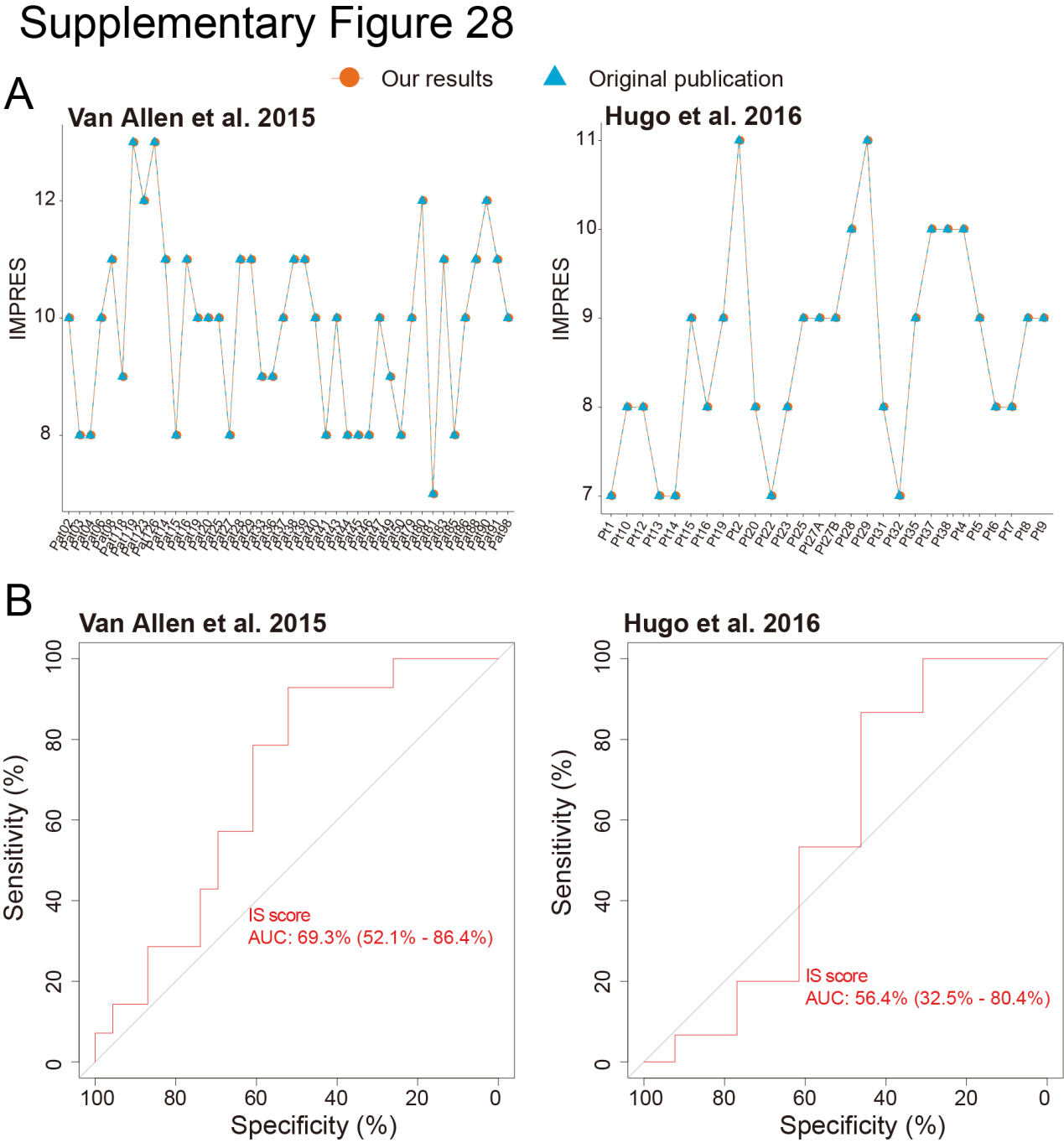


**Figure S1.** Accuracy assessment of the reconstructed calculation models. (**A**) Our reconstructed calculation models for IMPRES was highly consistent with original article using the same datasets. (**B**) Receiver operating characteristics (ROC) analysis of recalculated IS scores in the Van Allen et al. 2015 and the Hugo et al. 2016 datasets as original publication did.


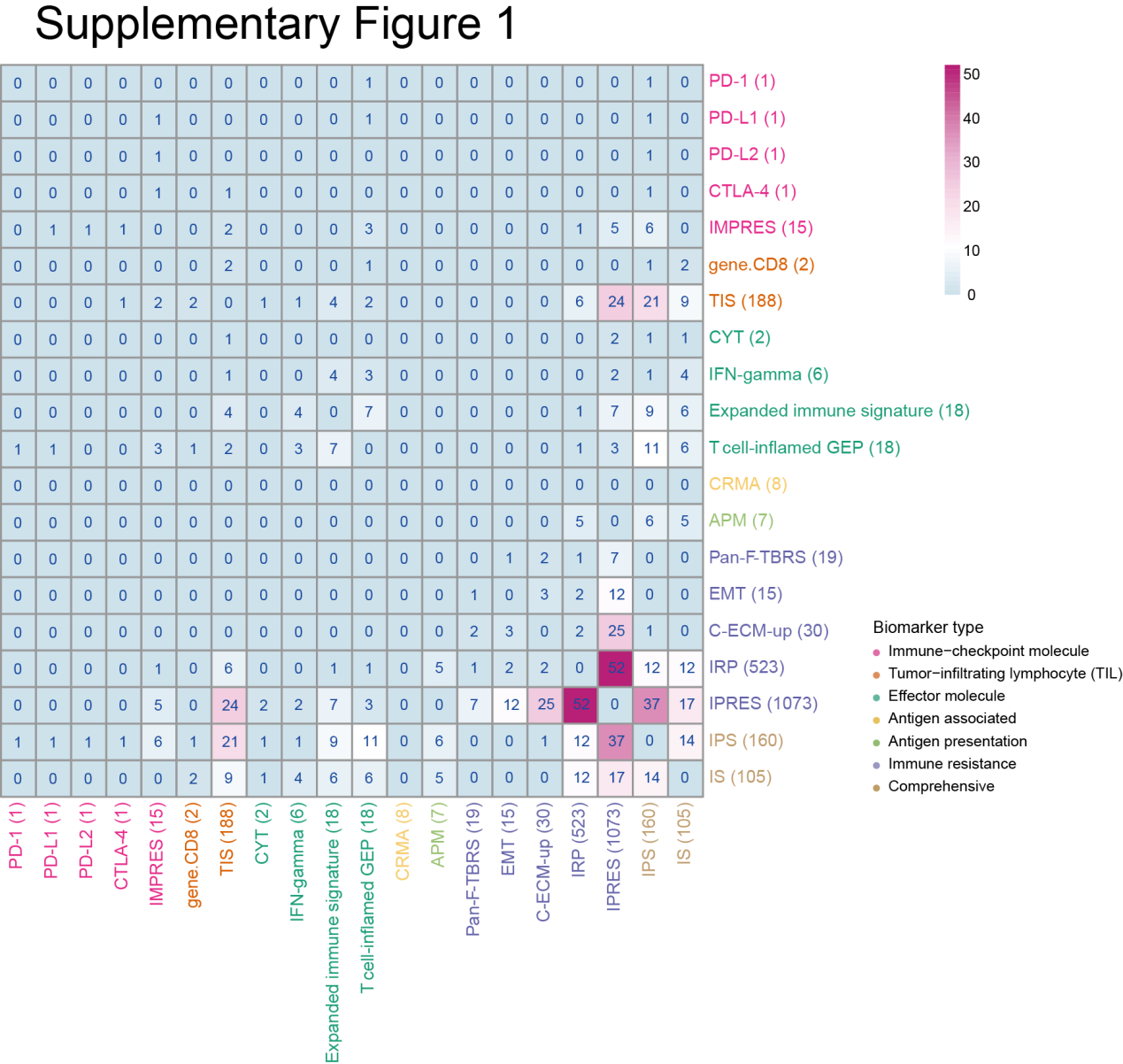


**Figure S2.** Evaluation of overlapped genes sharing between the transcriptomic biomarkers. Heatmap showed the number of overlapped genes between any pair of transcriptomic biomarkers. Darker red indicated more overlapped genes. The number of gene in each biomarker was shown in bracket.


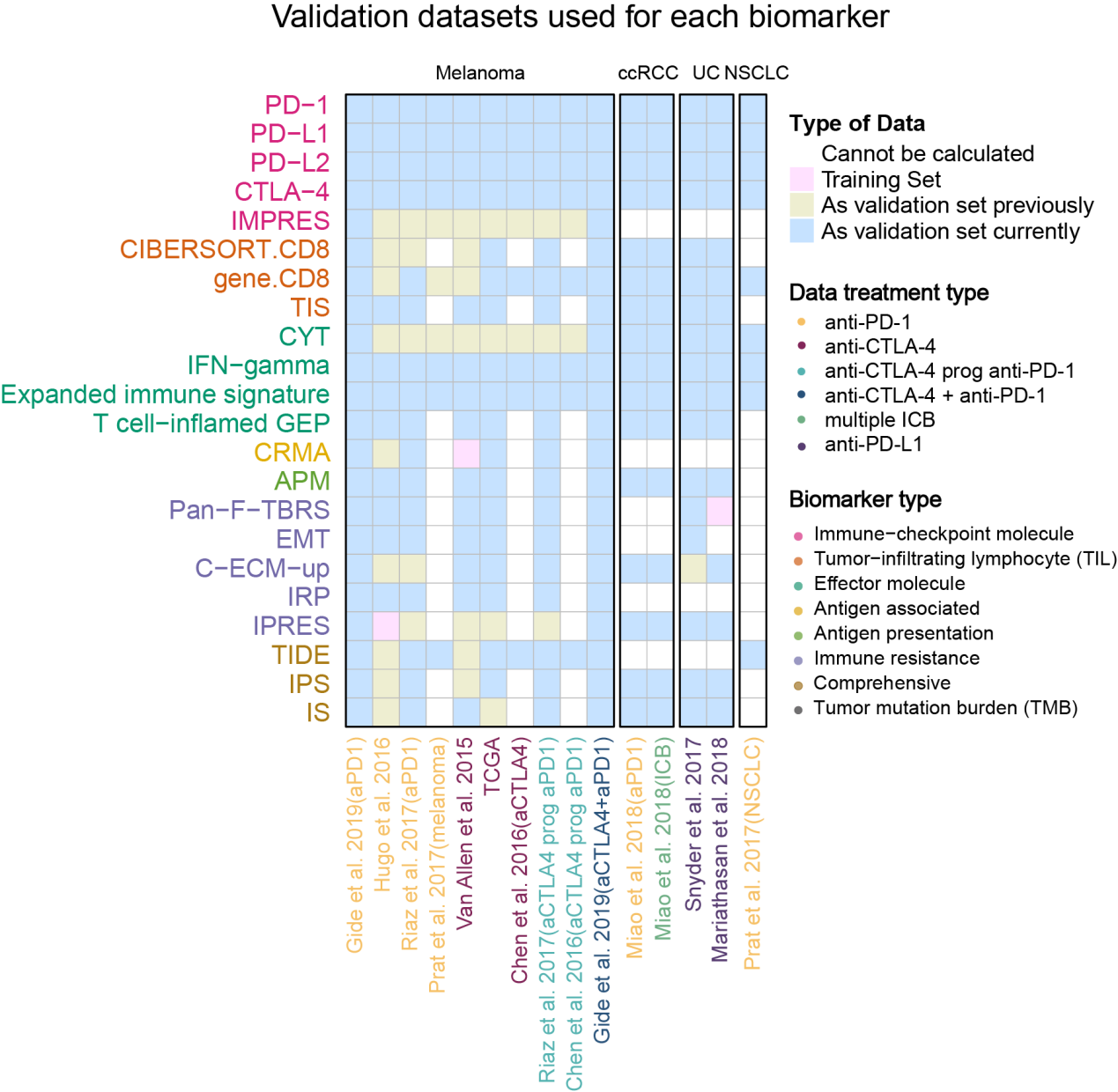


**Figure S3.** Validation datasets used for evaluation of each biomarker. The color indicated the way of data using. "Cannot be calculated" meaned the dataset didn't support calculating this biomarker. "Training Set" meaned the dataset was the training dataset used for biomarker identification. "As validation set previously" meaned the dataset had been used as independent validation data previously. "As validation set currently" meaned the dataset was being used as independent validation data in this study.


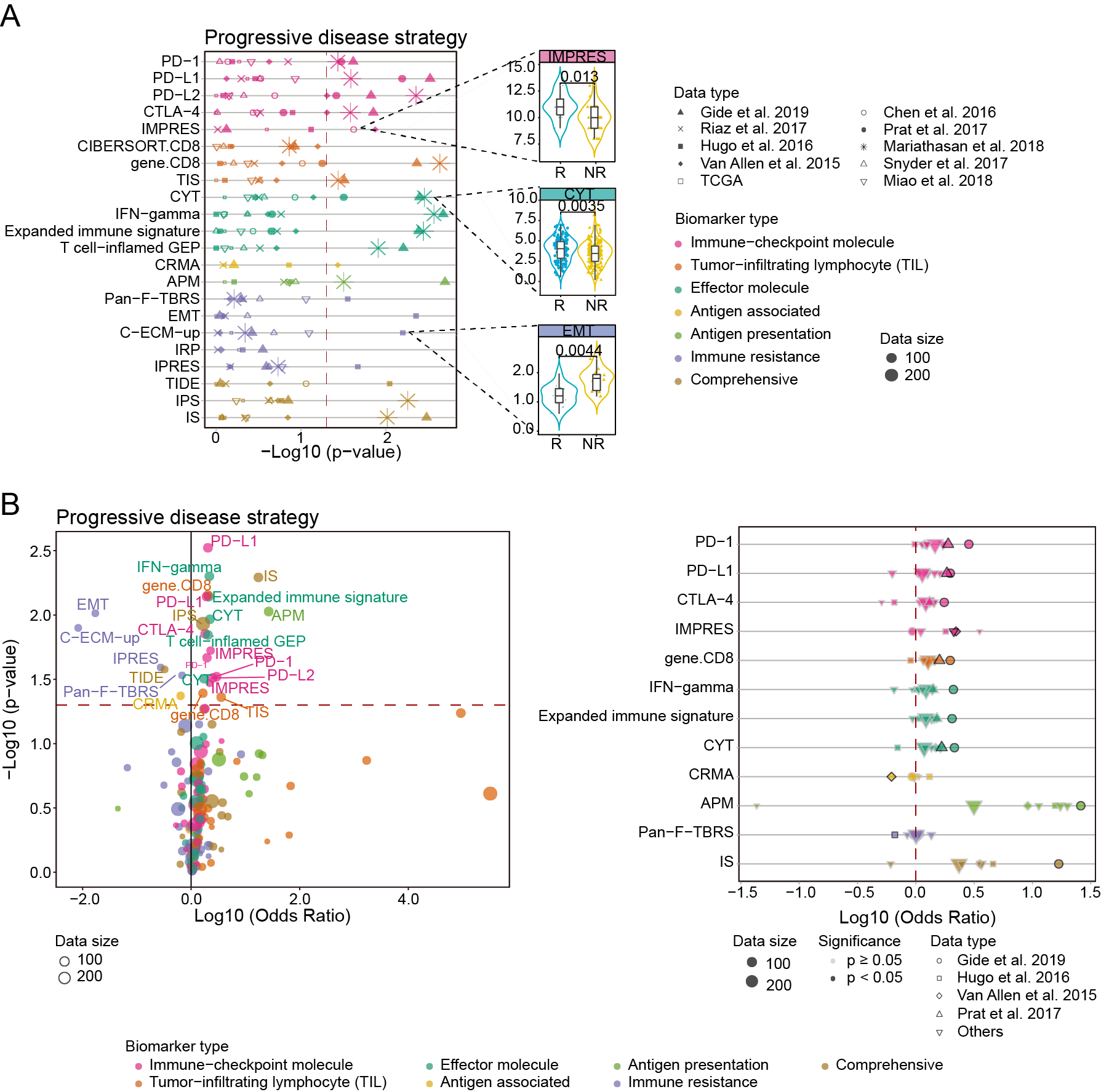


**Figure S4.** Association assessment between transcriptomic biomarkers and ICB clinical response under response classification strategy "PD" **at overall evaluation level.** (**A**) Left: the two-sided Wilcoxon rank-sum test *p* value indicating whether biomarkers significantly differentiate between responders (R) versus non-responders (NR) (patients stratification using "PD" strategy) in 10 benchmark datasets, red dashed line indicated 0.05 threshold of *p* value. Right: examples of biomarkers with significant difference (Wilcoxon rank-sum test *p* < 0.05) in score distribution between the responding (R) versus non-responding (NR) tumors. Black lines in the box represented upper 75%, median, and lower 25% values. (**B**) Left: Scatterplot showing −log10 (*p*-value) and log10 (Odds Ratio) from logistic regression model. Red dashed line indicated 0.05 threshold of *p* value. Right: Coefficients from logistic regression analysis of different biomarkers, the black edge of dots indicating significance level with *p* < 0.05.


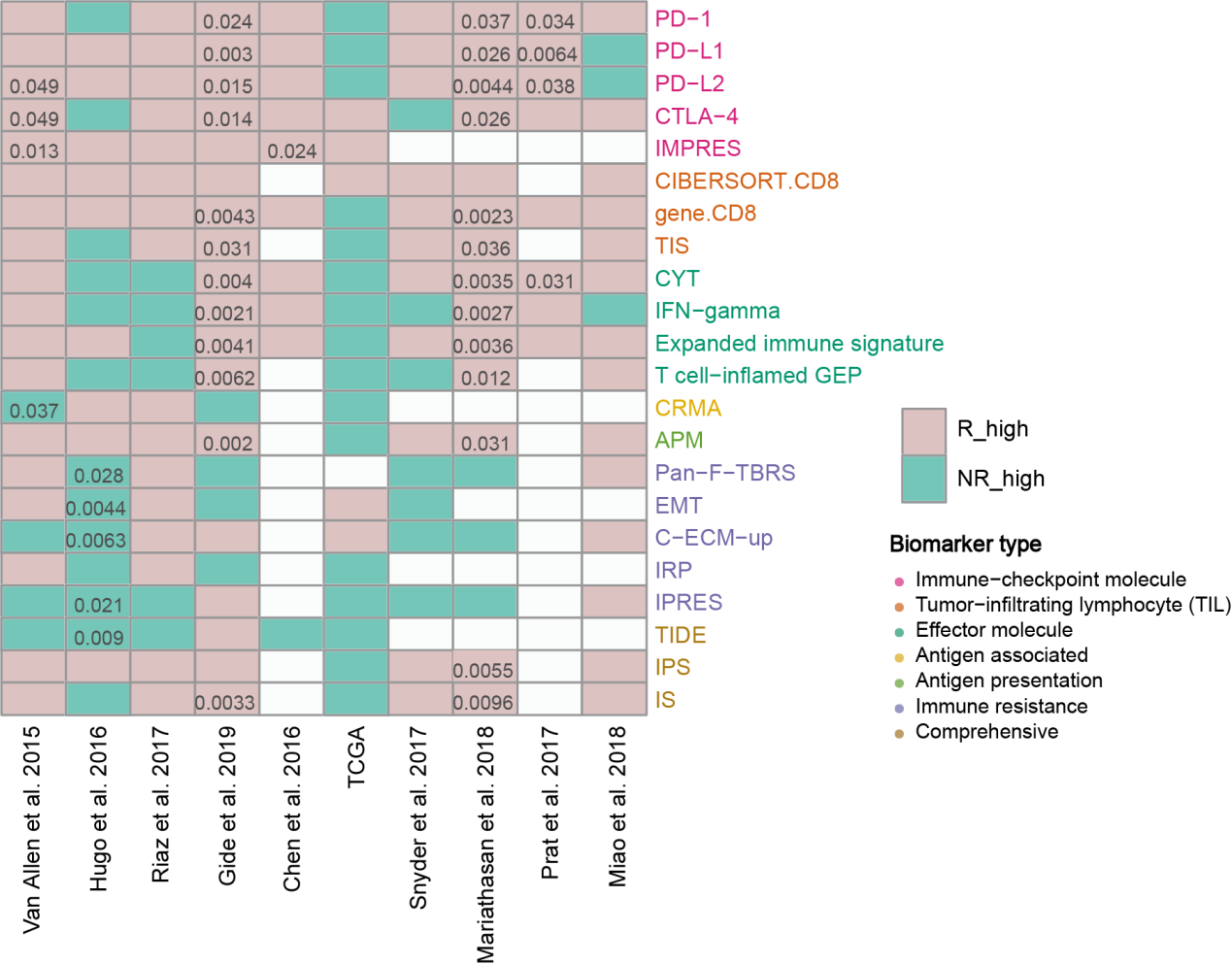


**Figure S5. (related to Figure S4). Correlation of each transcriptomic biomarker with ICB response at overall evaluation level.** The heatmap showed the association of 22 transcriptomic biomarkers with ICB responses under the response classification strategy "PD" in 10 benchmark datasets. Pink indicated that the median scores of the biomarkers in responders were higher than those in non-responders, green indicated that the median scores of the biomarkers in responders were lower than those in non-responder, and blank space indicated that the biomarkers cannot be scored in the dataset. Numbers in the square were the *p* value calculated via the two-sided Wilcoxon rank-sum test (only significant *p*-value results were given).


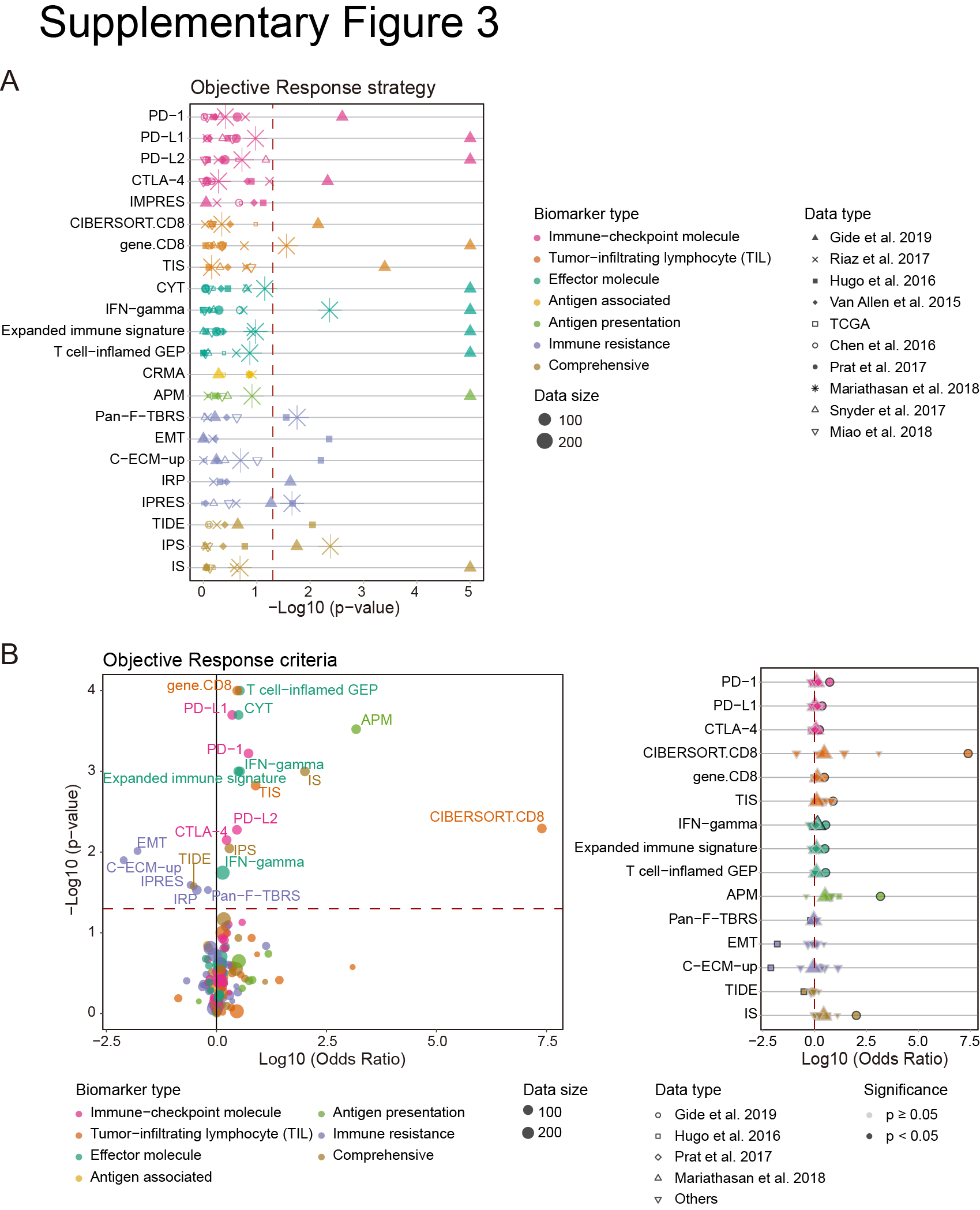


**Figure S6.** (related to Figure S4). Association assessment between transcriptomic biomarkers and ICB clinical response under response classification strategy "OR" **at overall evaluation level**. (**A**) Two-sided Wilcoxon rank-sum test *p* value indicating whether biomarkers significantly differentiated between responders versus non-responders (patients stratification using "OR" strategy), red dashed line indicated 0.05 threshold of *p* value. (**B**) Left: Scatterplot showing −log10 (*p*-value) and log10 (Odds Ratio) from logistic regression model. Red dashed line indicated 0.05 threshold of *p* value. Right: Coefficients from logistic regression analysis of different biomarkers, the black edge of dots indicating significance level with *p* < 0.05.


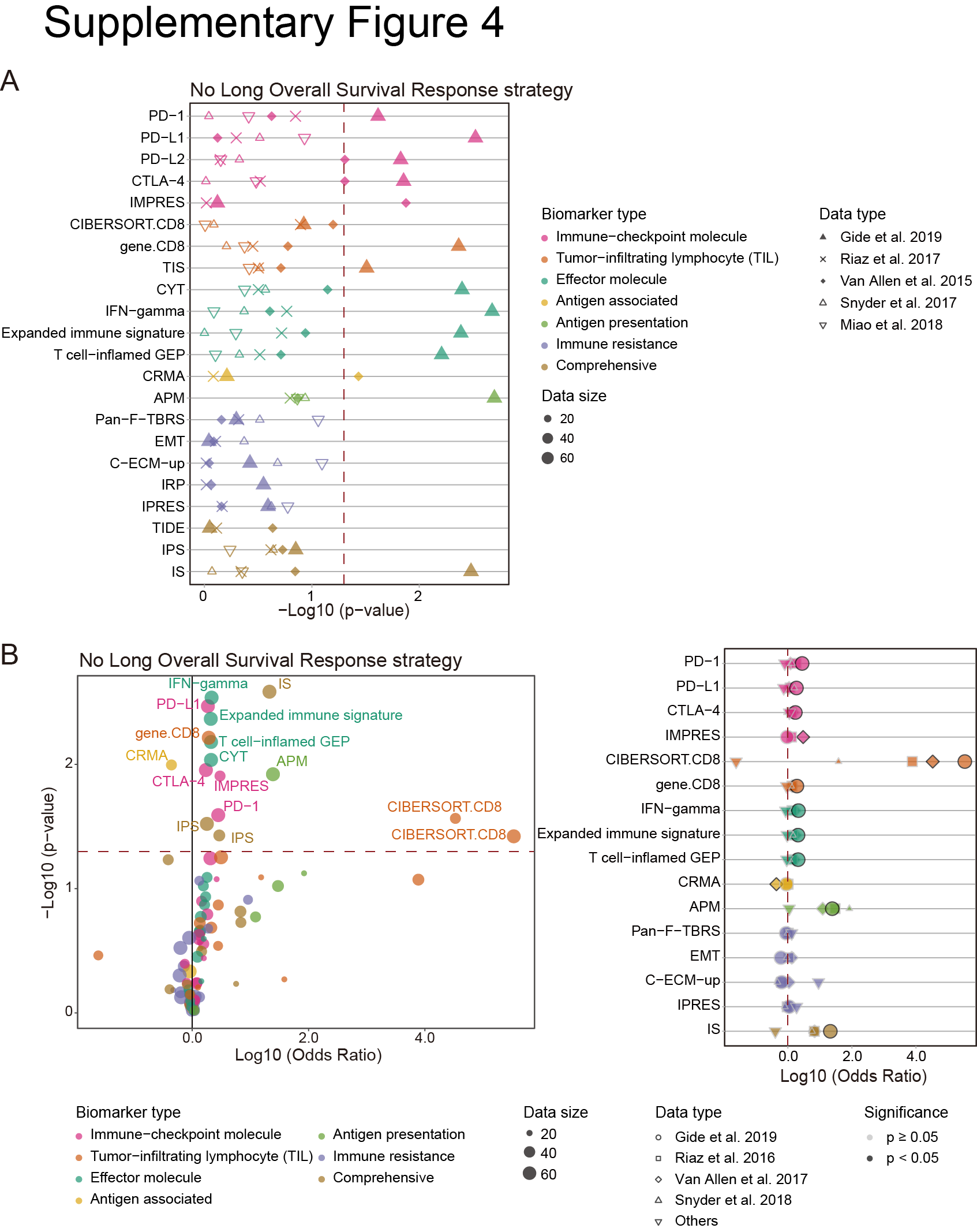


**Figure S7.** (related to Figure S4). Association assessment between transcriptomic biomarkers and ICB clinical response under response classification strategy "OS**" at overall evaluation level.** (**A**) Two-sided Wilcoxon rank-sum test *p* value indicating whether biomarkers significantly differentiated between responders versus non-responders (patients stratification using "OS" strategy), red dashed line indicated 0.05 threshold of *p* value. (**B**) Left: Scatterplot showing −log10 (*p*-value) and log10 (Odds Ratio) from logistic regression model. Red dashed line indicated 0.05 threshold of *p* value. Right: Coefficients from logistic regression analysis of different biomarkers, the black edge of dots indicating significance level with *p* < 0.05.


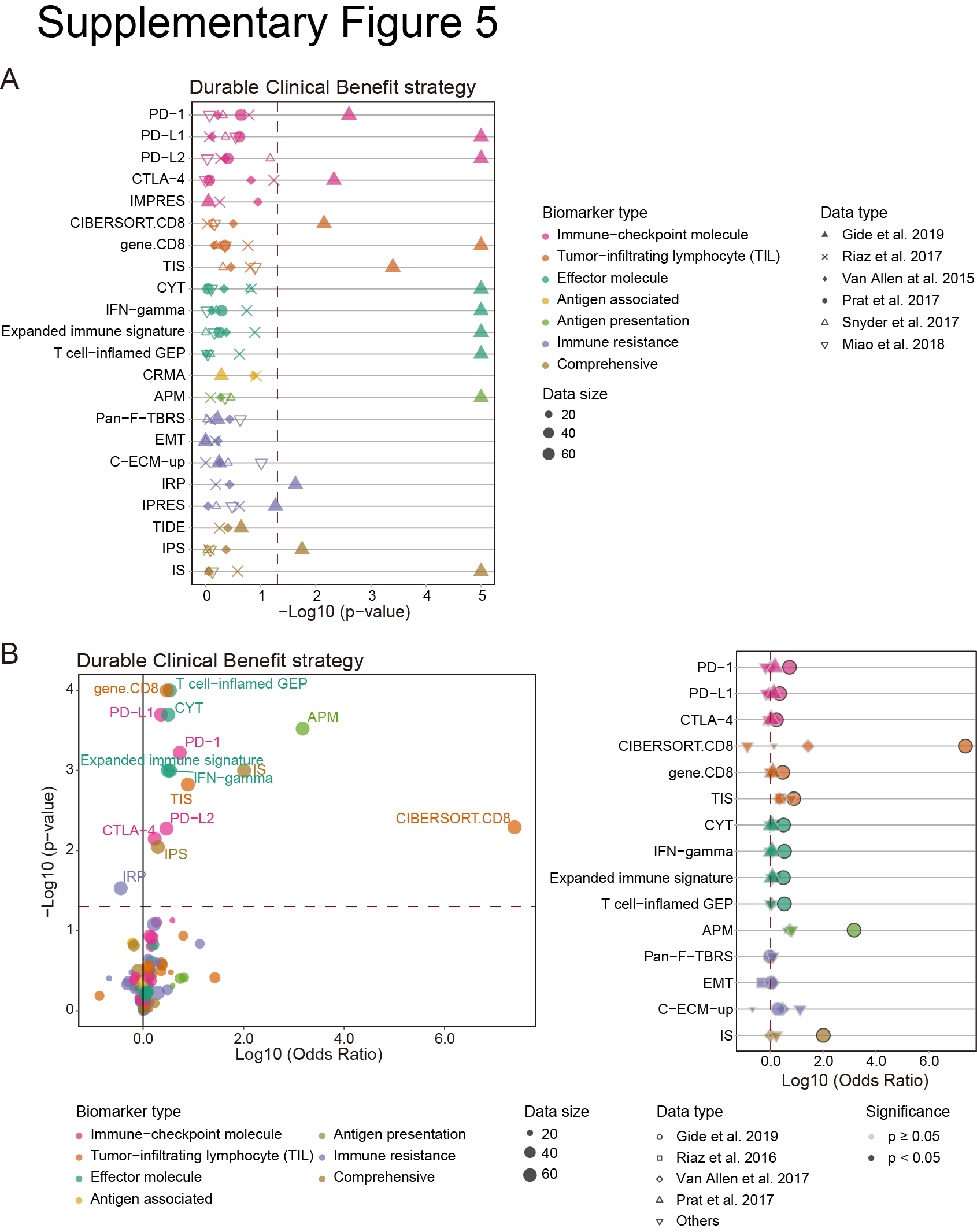


**Figure S8.** (related to Figure S4). Association assessment between transcriptomic biomarkers and ICB clinical response under response classification strategy "DCB" **at overall evaluation level**. (**A**) Two-sided Wilcoxon rank-sum test *p* value indicating whether biomarkers significantly differentiated between responders versus non-responders (patients stratification using "DCB" strategy), red dashed line indicated 0.05 threshold of *p* value. (**B**) Left: Scatterplot showing −log10 (*p*-value) and log10 (Odds Ratio) from logistic regression model. Red dashed line indicated 0.05 threshold of *p* value. Right: Coefficients from logistic regression analysis of different biomarkers, the black edge of dots indicating significance level with *p* < 0.05.


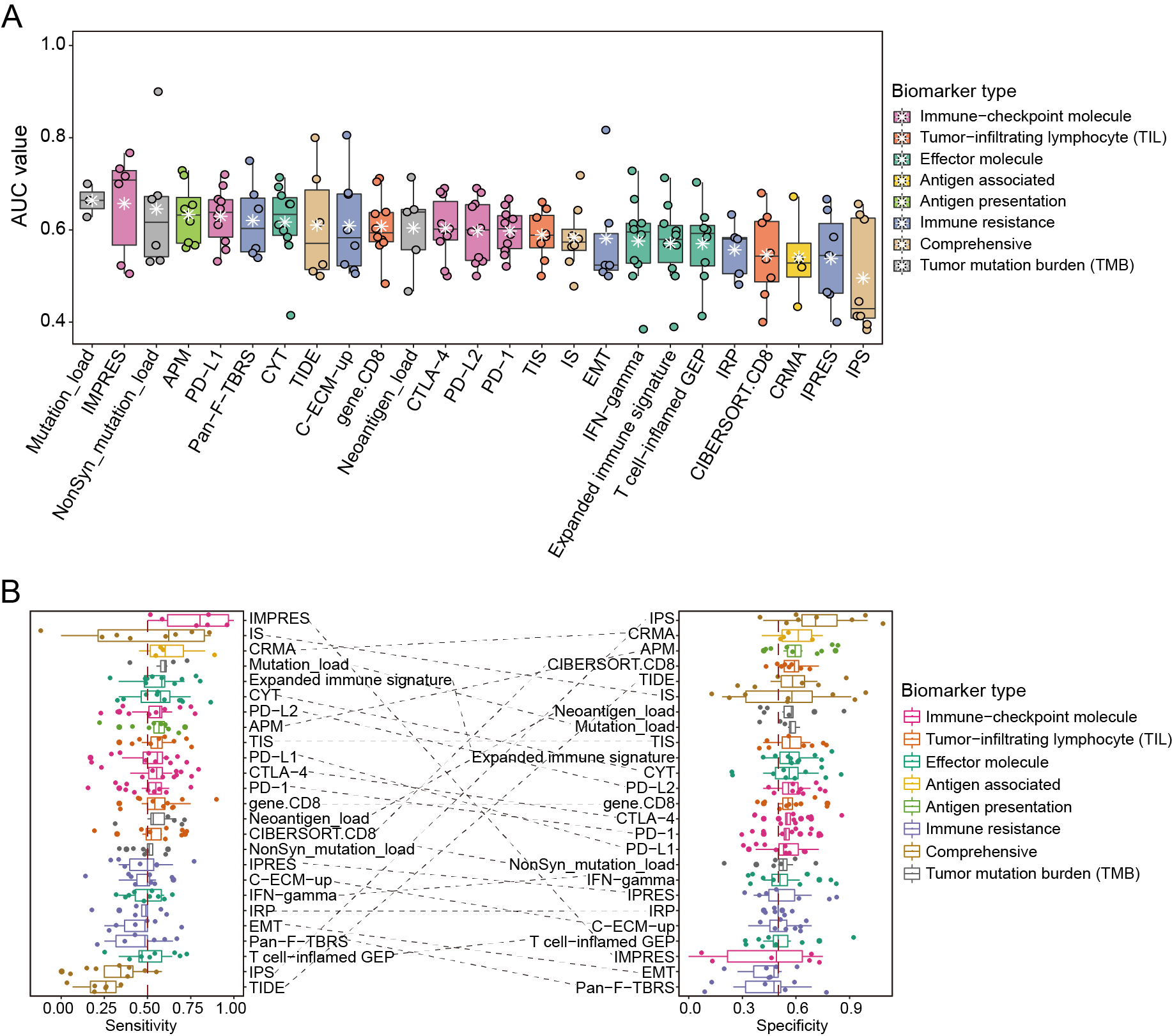


**Figure S9.** Prediction performance of biomarkers for ICB response at overall evaluation level. (**A**) The AUC values distribution of each biomarker under response classification strategies "PD" in 10 benchmark datasets. Black lines in the box represented upper 75%, median, and lower 25% values of biomarkers. Ordering the biomarkers according to their mean AUCs. For each biomarker, its training data was not included in the AUC calculation. (**B**) Sensitivity and specificity of each biomarker in 10 benchmark datasets calculated by the median value as the threshold to make binary predictions.


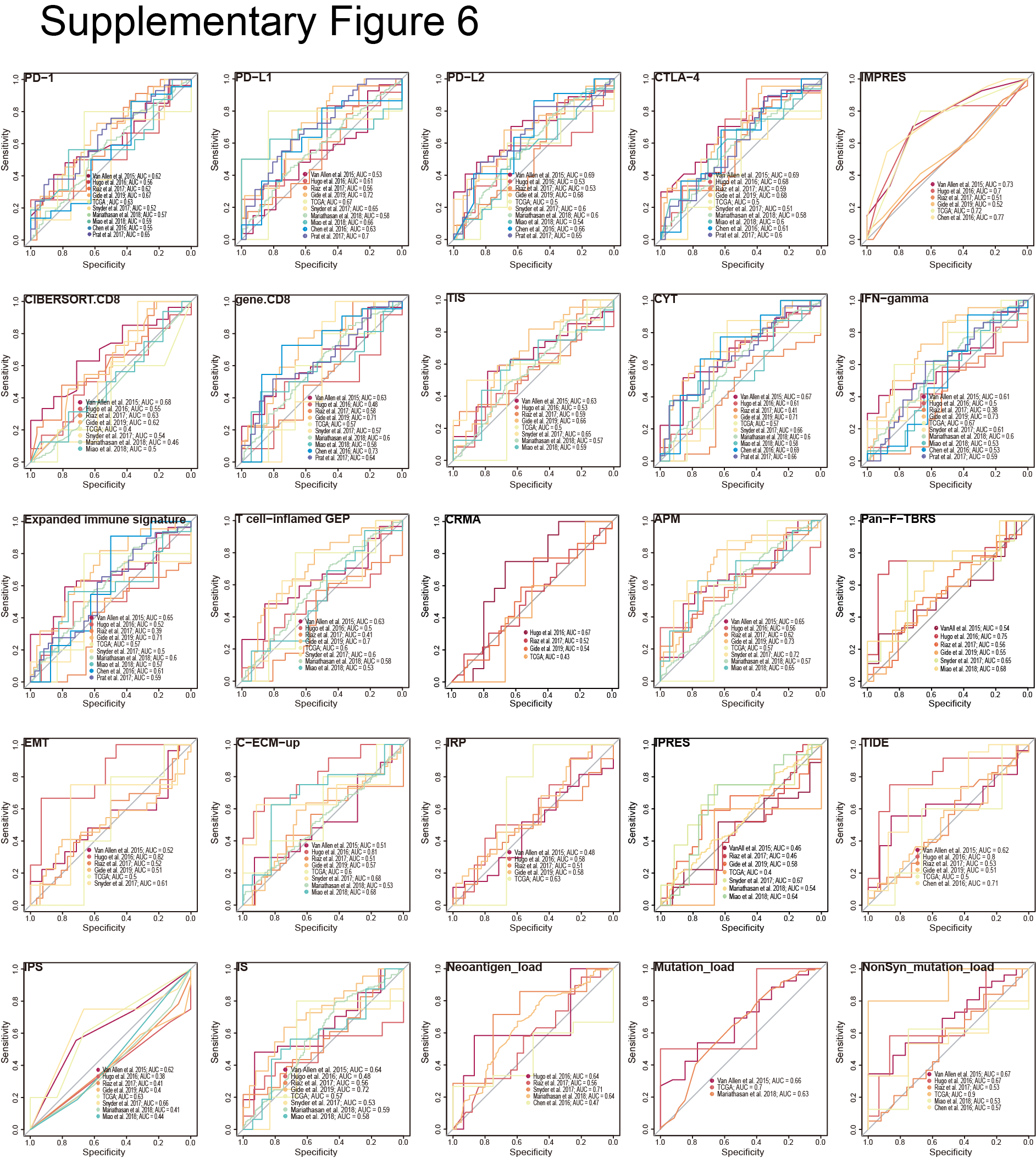


**Figure S10.** (related to Figure S9). Prediction performance of each biomarker for ICB response **at overall evaluation level**. ROC curves quantifying the prediction accuracy of different biomarkers across 10 benchmark datasets.


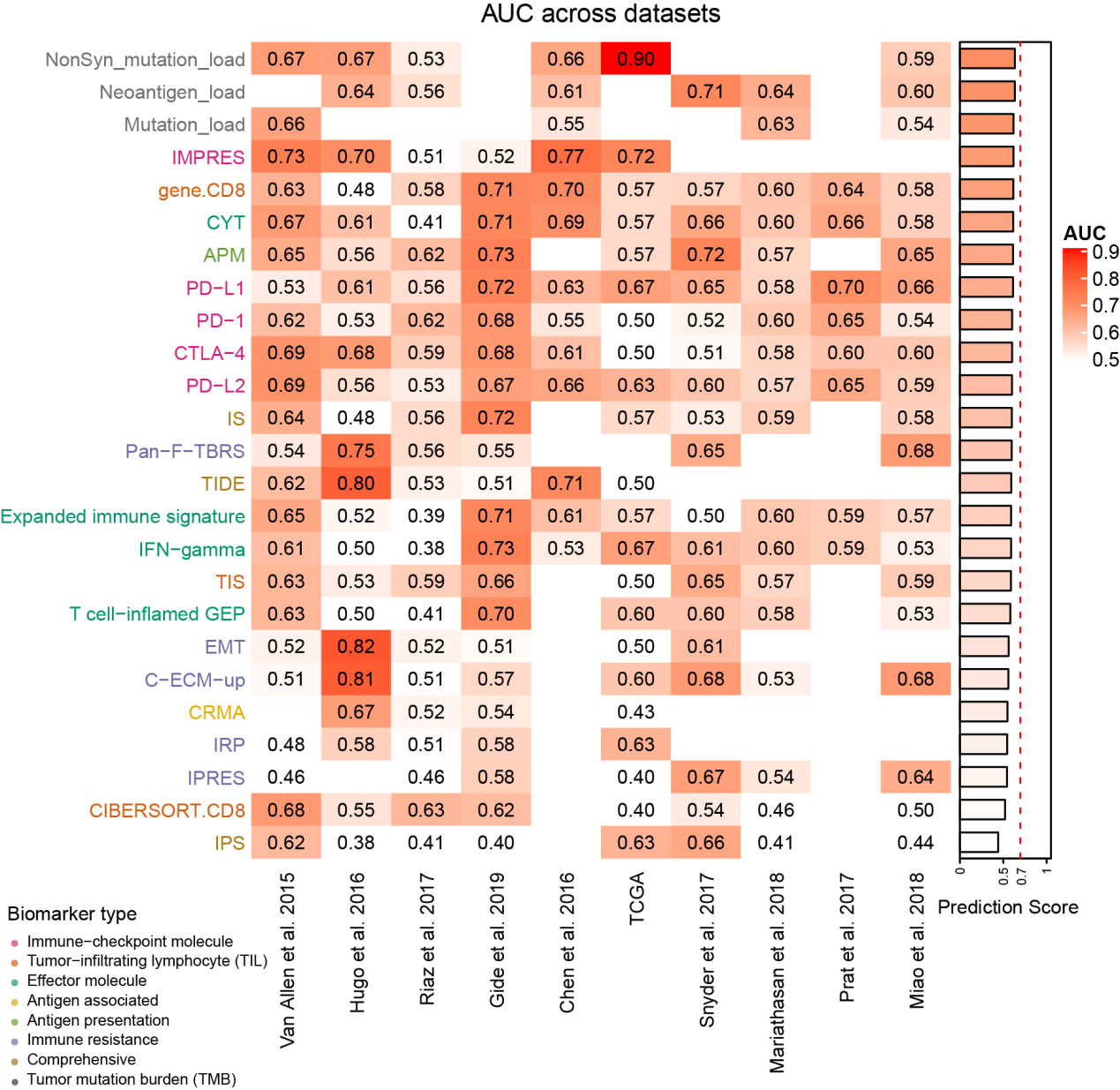


**Figure S11.** (related to Figure S10). Prediction performance of each biomarker for ICB response at overall evaluation level. AUC across 10 benchmark datasets and the Prediction Score (sum of sample size-weighted AUC) of different biomarkers were shown.


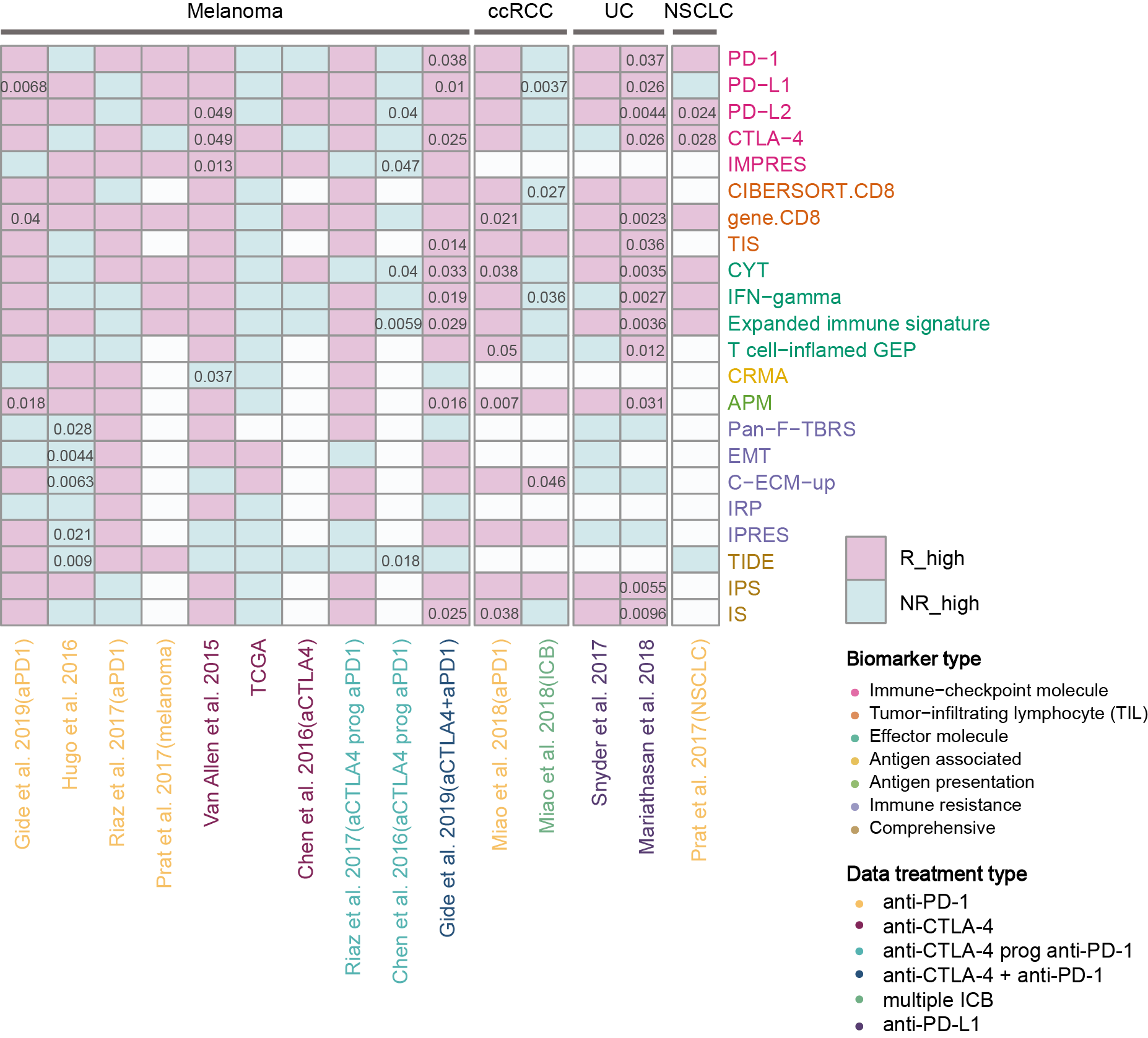


**Figure S12.** (related to Figure 3 and Figure 4). Correlation of each transcriptomic biomarker with ICB response in melanoma, ccRCC, UC and NSCLC. The heatmap showed the association of 22 transcriptomic biomarkers with ICB responses under the response classification strategy "PD" in 15 benchmark datasets with 4 cancer type. Rose-red indicated that the median scores of biomarkers in responders were higher than those in non-responders, blue indicated that the median scores of biomarkers in responders were lower than those in non-responders, and blank space indicated that the biomarkers cannot be scored in the dataset. Numbers in the square were the *p* value calculated via the two-sided Wilcoxon rank-sum test (only significant *p*-value results were given). The datasets with different ICB treatments were represented by different colors.


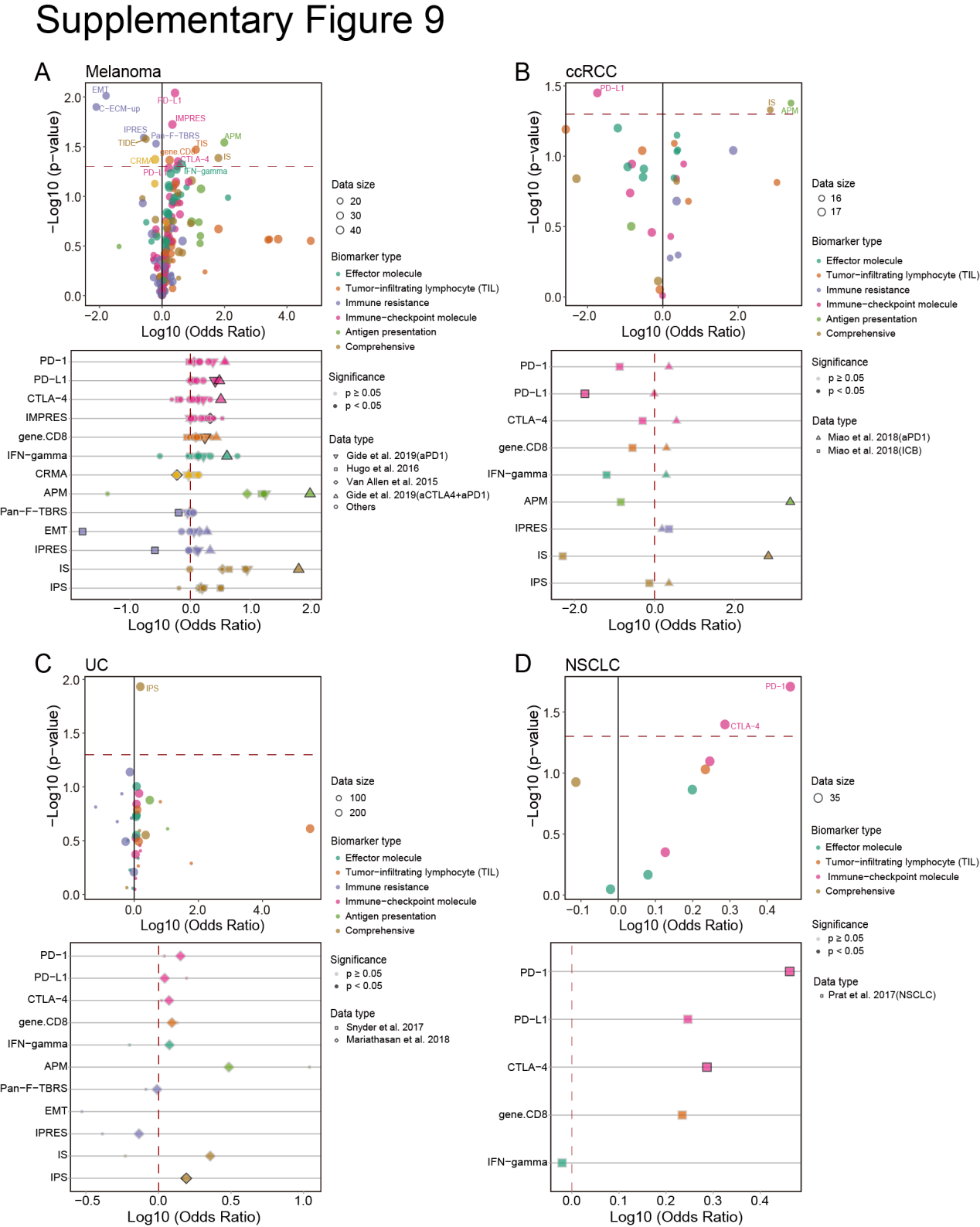


**Figure S13.** (related to Figure 3). Association assessment between transcriptomic biomarkers and ICB clinical response under response classification strategy "PD" across different cancer types. Response association assessment (patients stratification using "PD" strategy) results from logistic regression model in the melanoma datasets (**A**), ccRCC datasets (**B**), UC datasets (**C**) and NSCLC dataset (**D**). Scatterplot showing −log10 (*p*-value) and log10 (Odds Ratio). Black edge of dots indicating significance level with *p* < 0.05.


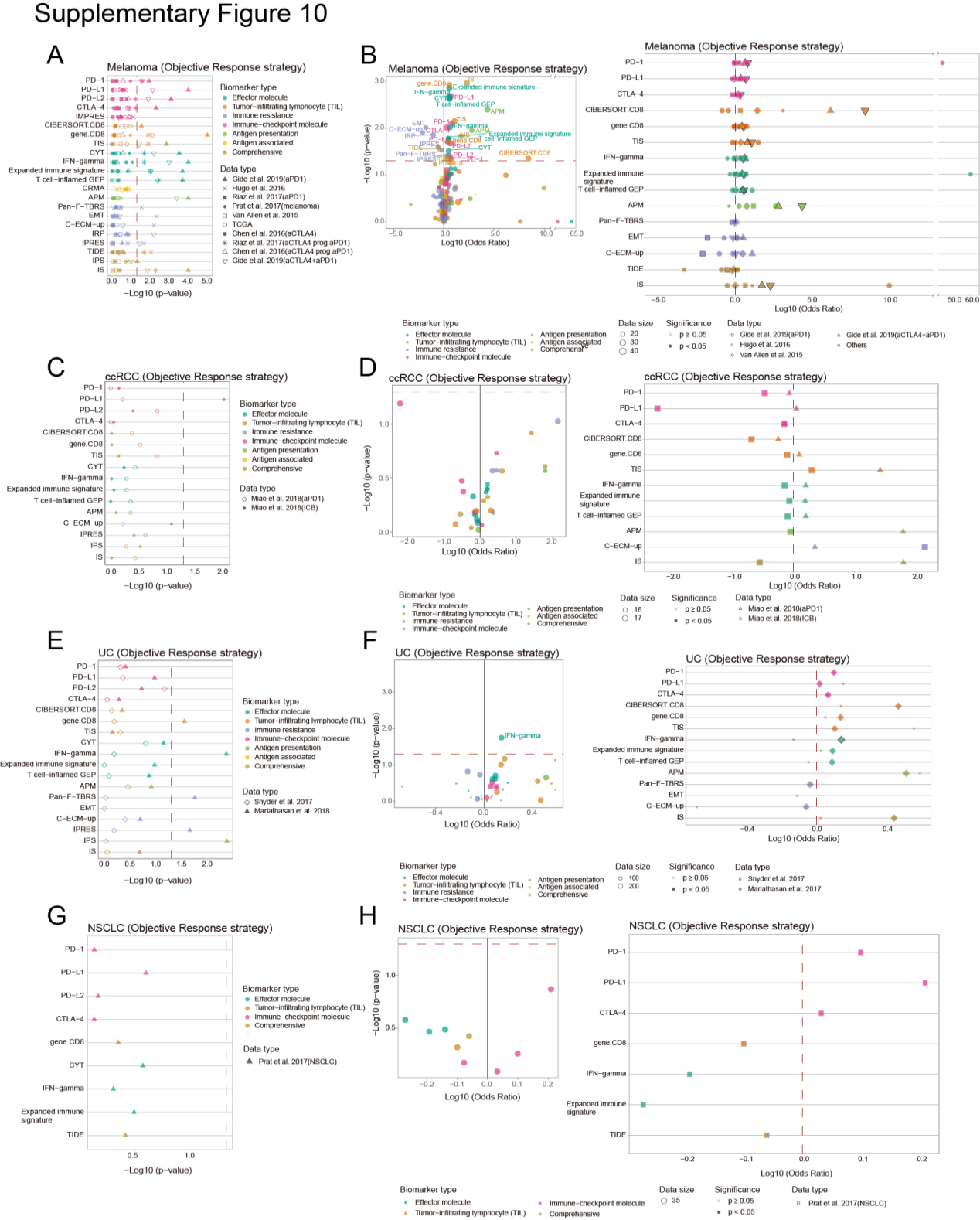


**Figure S14.** (related to Figure 3). Association assessment between transcriptomic biomarkers and ICB clinical response under response classification strategy "OR" across different cancer types. Two-sided Wilcoxon rank-sum test *p* value indicating whether biomarkers significantly differentiated between responders versus non-responders (patients stratification using "OR" strategy) in melanoma datasets (**A**), ccRCC datasets (**C**), UC datasets (**E**) and NSCLC dataset (**G**). Red dashed line indicated 0.05 threshold of *p* value. Scatterplot showing −log10 (*p*-value) and log10 (Odds Ratio) from logistic regression model in melanoma datasets (**B**), ccRCC datasets (**D**), UC datasets (**F**) and NSCLC dataset (**H**). Red dashed line indicated 0.05 threshold of *p* value. The black edge of dots indicating significance level with *p* < 0.05.


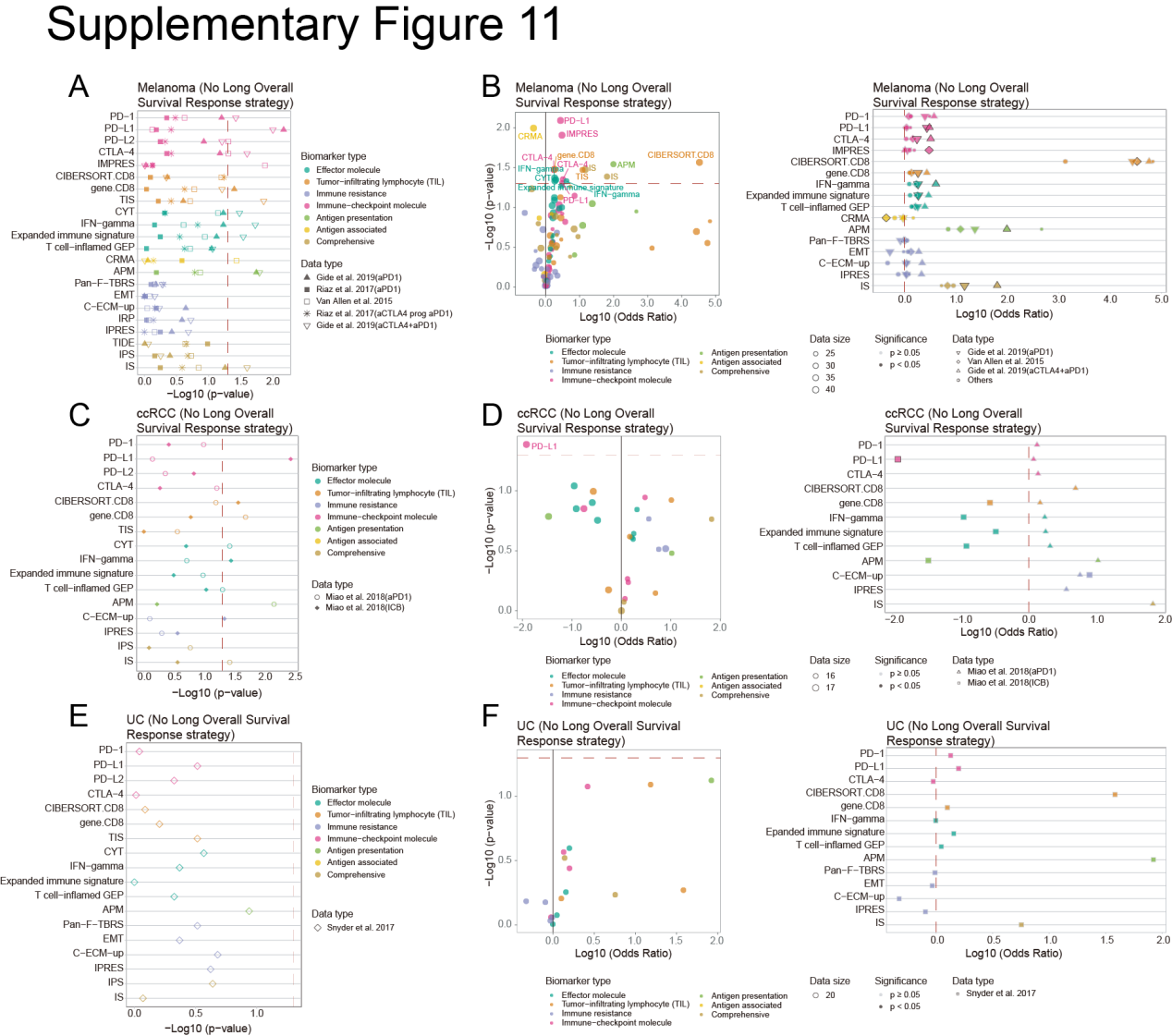


**Figure S15.** (related to Figure 3). Association assessment between transcriptomic biomarkers and ICB clinical response under response classification strategy "OS" across different cancer types. Two-sided Wilcoxon rank-sum test *p* value indicating whether biomarkers significantly differentiated between responders versus non-responders (patients stratification using "OS" strategy) in melanoma datasets (**A**), ccRCC datasets (**C**) and UC dataset (**E**). Red dashed line indicated 0.05 threshold of *p* value. Scatterplot showing −log10 (*p*-value) and log10 (Odds Ratio) from logistic regression model in melanoma datasets (**B**), ccRCC datasets (**D**) and UC dataset (**F**). Red dashed line indicated 0.05 threshold of *p* value. The black edge of dots indicating significance level with *p* < 0.05. NSCLC dataset was missing due to the lack of overall survival data.


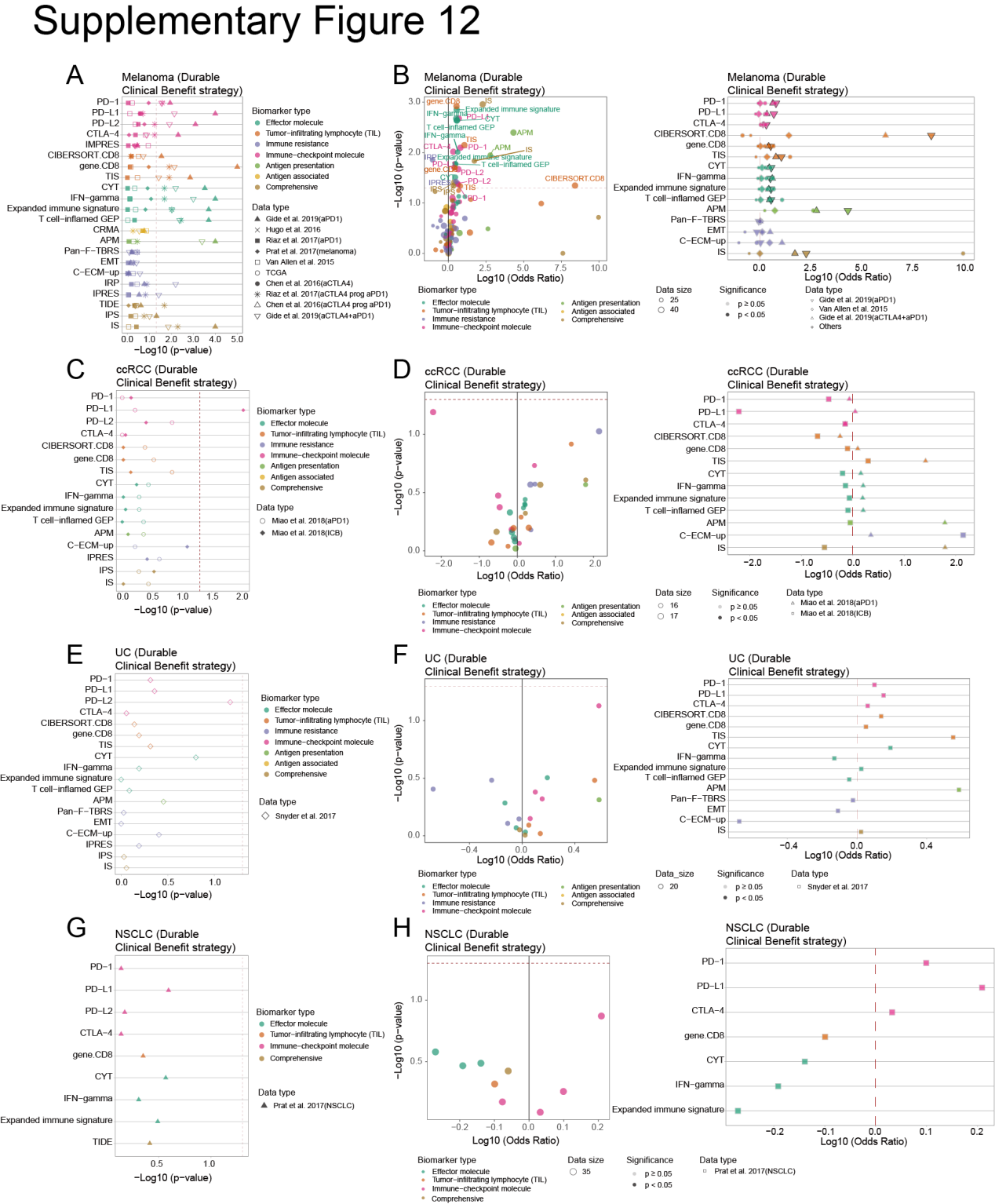


**Figure S16.** (related to Figure 3). Association assessment between transcriptomic biomarkers and ICB clinical response under response classification strategy "DCB" across different cancer types. Two-sided Wilcoxon rank-sum test *p* value indicating whether biomarkers significantly differentiated between responders versus non-responders (patients stratification using "DCB" strategy) in melanoma datasets (**A**), ccRCC datasets (**C**), UC dataset (**E**) and NSCLC dataset (**G**). Red dashed line indicated 0.05 threshold of *p* value. Scatterplot showing −log10 (*p*-value) and log10 (Odds Ratio) from logistic regression model in melanoma datasets (**B**), ccRCC datasets (**D**), UC dataset (**F**) and NSCLC dataset (**H**). Red dashed line indicated 0.05 threshold of *p* value. The black edge of dots indicating significance level with *p* < 0.05.


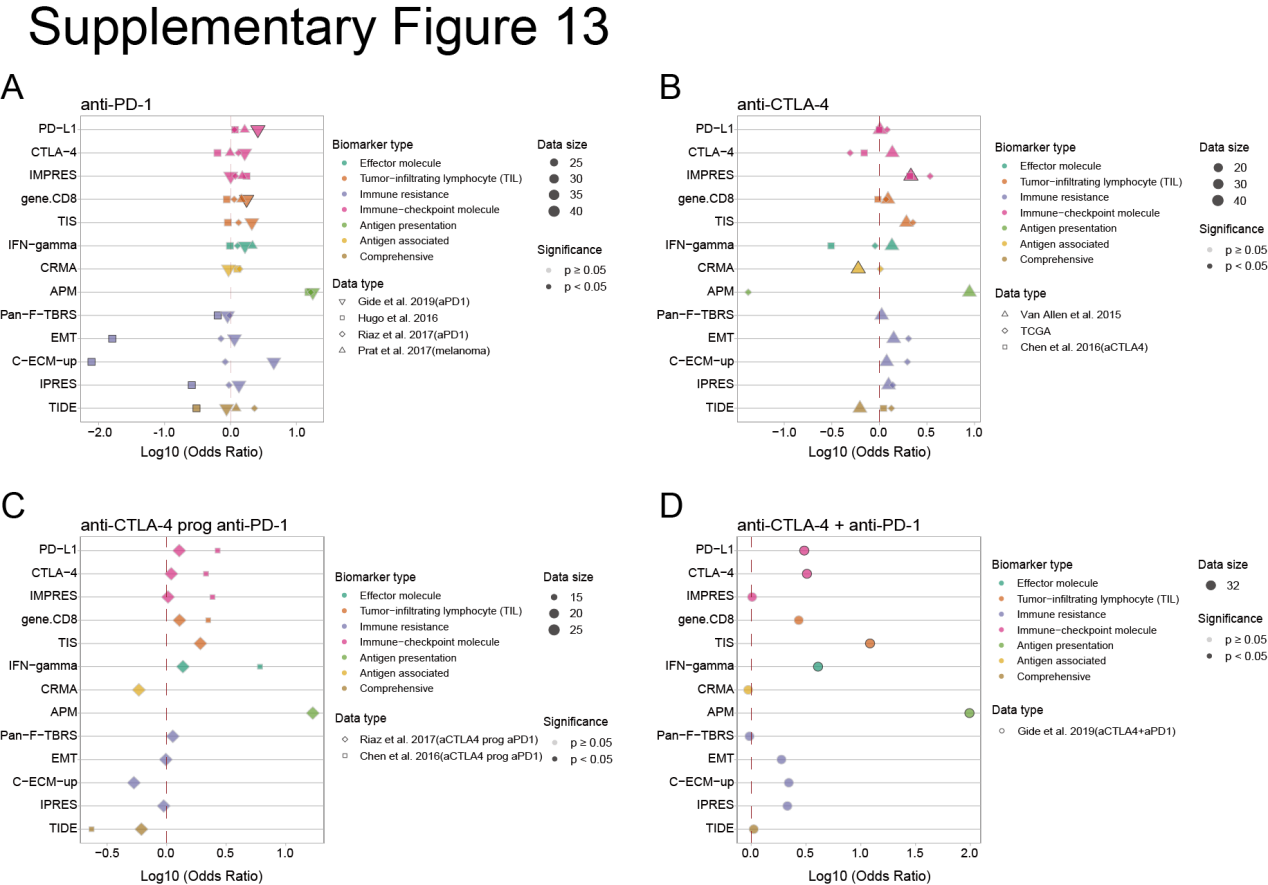


**Figure S17.** (related to Figure 4). Association assessment between transcriptomic biomarkers and ICB clinical response under response classification strategy "PD" across different ICB therapy strategies. Coefficients from logistic regression analysis stratified by different biomarkers in anti-PD-1 therapy (**A**), anti-CTLA-4 therapy (**B**), anti-CTLA-4 prog anti-PD-1 therapy (**C**) and the combination therapy of anti-CTLA-4 and anti-PD-1 (**D**). The black edge of dots indicating significance level with *p* < 0.05.


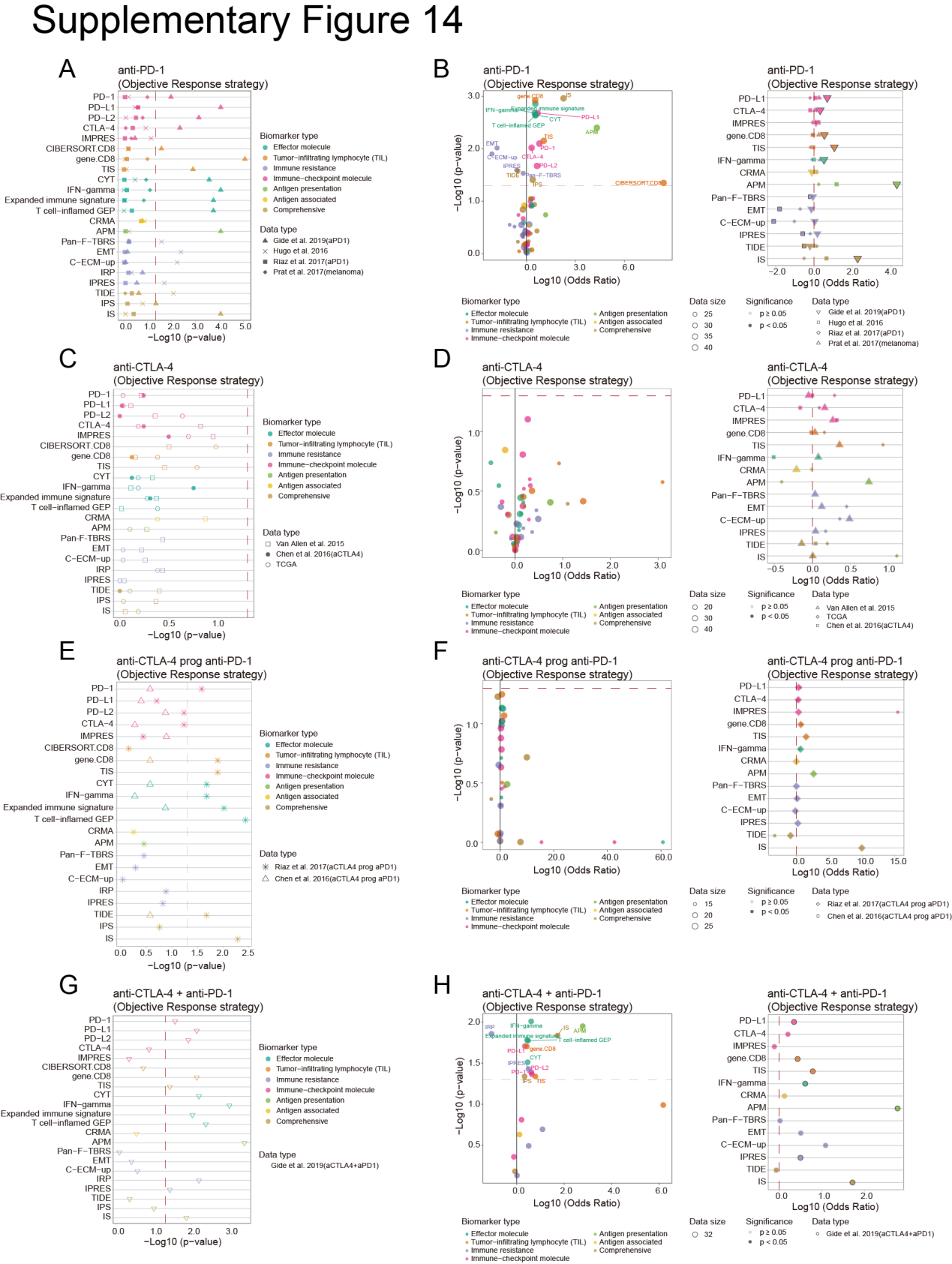


**Figure S18.** (related to Figure 4). Association assessment between transcriptomic biomarkers and ICB clinical response under response classification strategy "OR" across different ICB therapy strategies. Two-sided Wilcoxon rank-sum test *p* value indicating whether biomarkers significantly differentiated between responders versus non-responders (patients stratification using "OR" strategy) in anti-PD-1 datasets (**A**), anti-CTLA-4 datasets (**C**), anti-CTLA-4 prog anti-PD-1 datasets (**E**) and combination therapy dataset (**G**). Red dashed line indicated 0.05 threshold of *p* value. Scatterplot showing −log10 (*p*-value) and log10 (Odds Ratio) from logistic regression model in anti-PD-1 datasets (**B**), anti-CTLA-4 datasets (**D**), anti-CTLA-4 prog anti-PD-1 datasets (**F**) and combination therapy dataset (H). Red dashed line indicated 0.05 threshold of *p* value. The black edge of dots indicating significance level with *p* < 0.05.


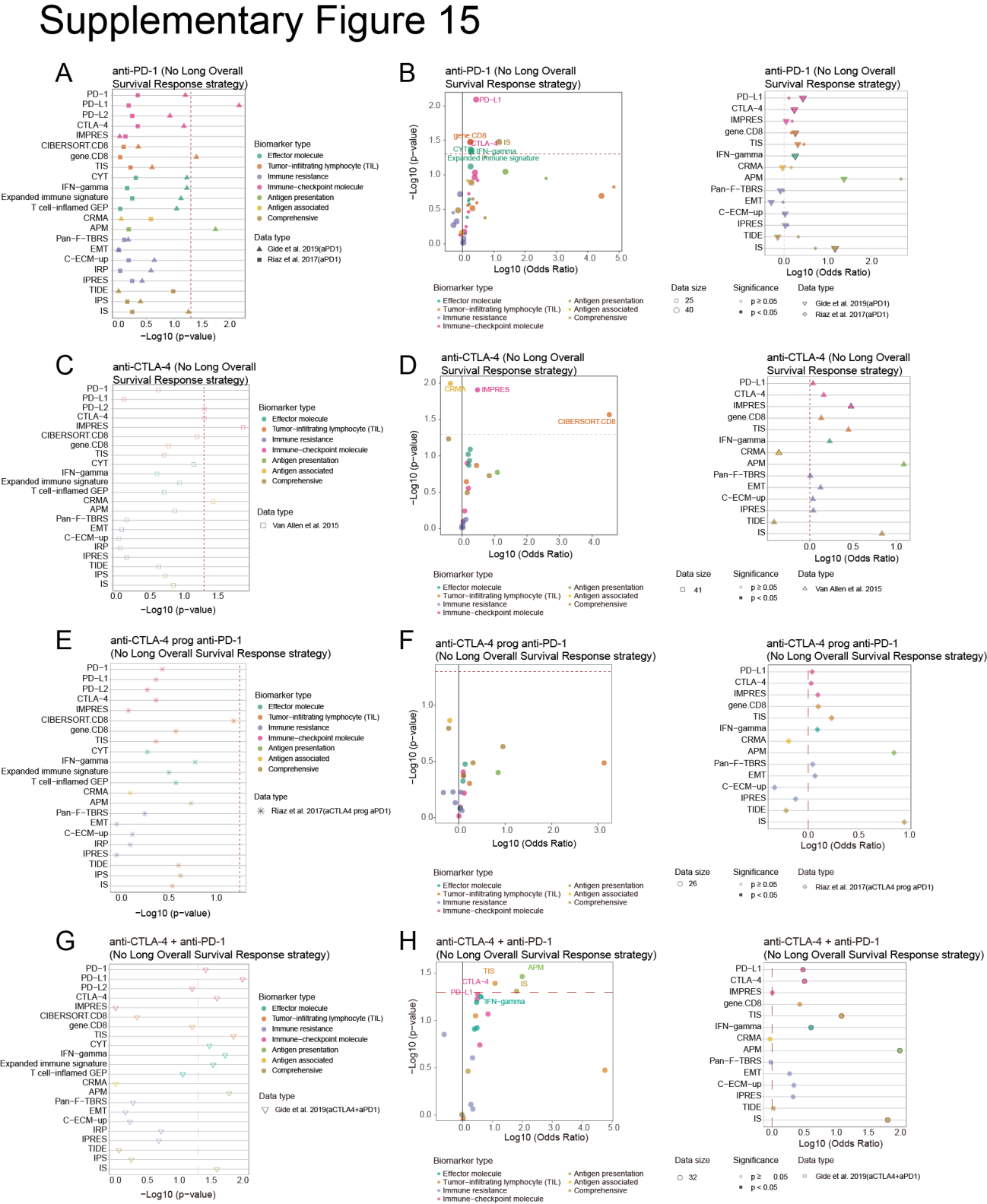


**Figure S19.** (related to Figure 4). Association assessment between transcriptomic biomarkers and ICB clinical response under response classification strategy "OS" across different ICB therapy strategies. Two-sided Wilcoxon rank-sum test *p* value indicating whether biomarkers significantly differentiated between responders versus non-responders (patients stratification using "OS" strategy) in anti-PD-1 datasets (**A**), anti-CTLA-4 datasets (**C**), anti-CTLA-4 prog anti-PD-1 dataset (**E**) and combination therapy dataset (**G**). Red dashed line indicated 0.05 threshold of *p* value. Scatterplot showing −log10 (*p*-value) and log10 (Odds Ratio) from logistic regression model in anti-PD-1 datasets (**B**), anti-CTLA-4 datasets (**D**), anti-CTLA-4 prog anti-PD-1 dataset (**F**) and combination therapy dataset (**H**). Red dashed line indicated 0.05 threshold of *p* value. The black edge of dots indicating significance level with *p* < 0.05.


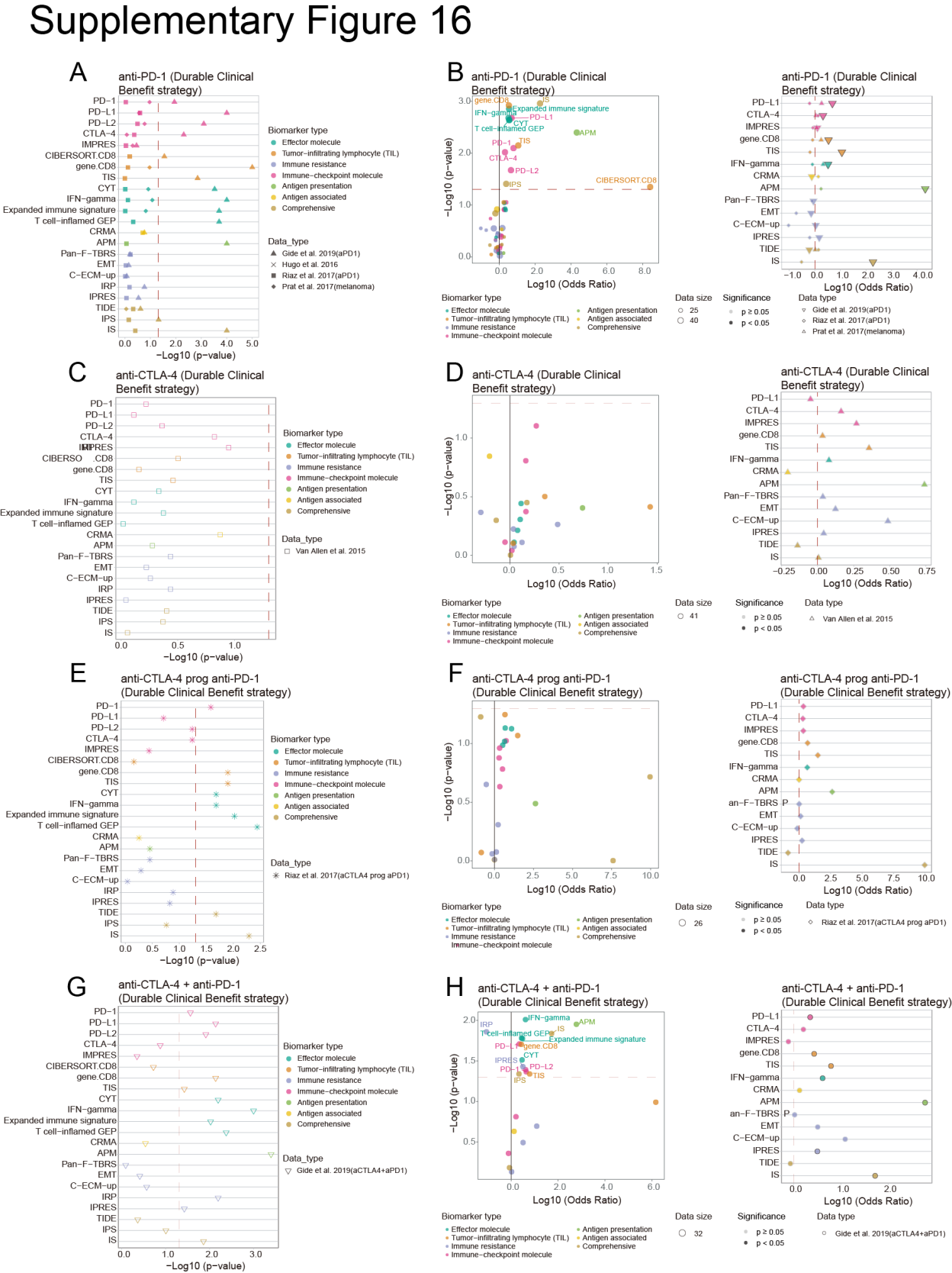


**Figure S20.** (related to Figure 4). Association assessment between transcriptomic biomarkers and ICB clinical response under response classification strategy "DCB" across different ICB therapy strategies. Two-sided Wilcoxon rank-sum test *p* value indicating whether biomarkers significantly differentiated between responders versus non-responders (patients stratification using "DCB" strategy) in anti-PD-1 datasets (**A**), anti-CTLA-4 dataset (**C**), anti-CTLA-4 prog anti-PD-1 dataset (**E**) and combination therapy dataset (**G**). Red dashed line indicated 0.05 threshold of *p* value. Scatterplot showing −log10 (*p*-value) and log10 (Odds Ratio) from logistic regression model in anti-PD-1 datasets (**B**), anti-CTLA-4 dataset (**D**), anti-CTLA-4 prog anti-PD-1 dataset (**F**) and combination therapy dataset (H). Red dashed line indicated 0.05 threshold of *p* value. The black edge of dots indicating significance level with *p* < 0.05.


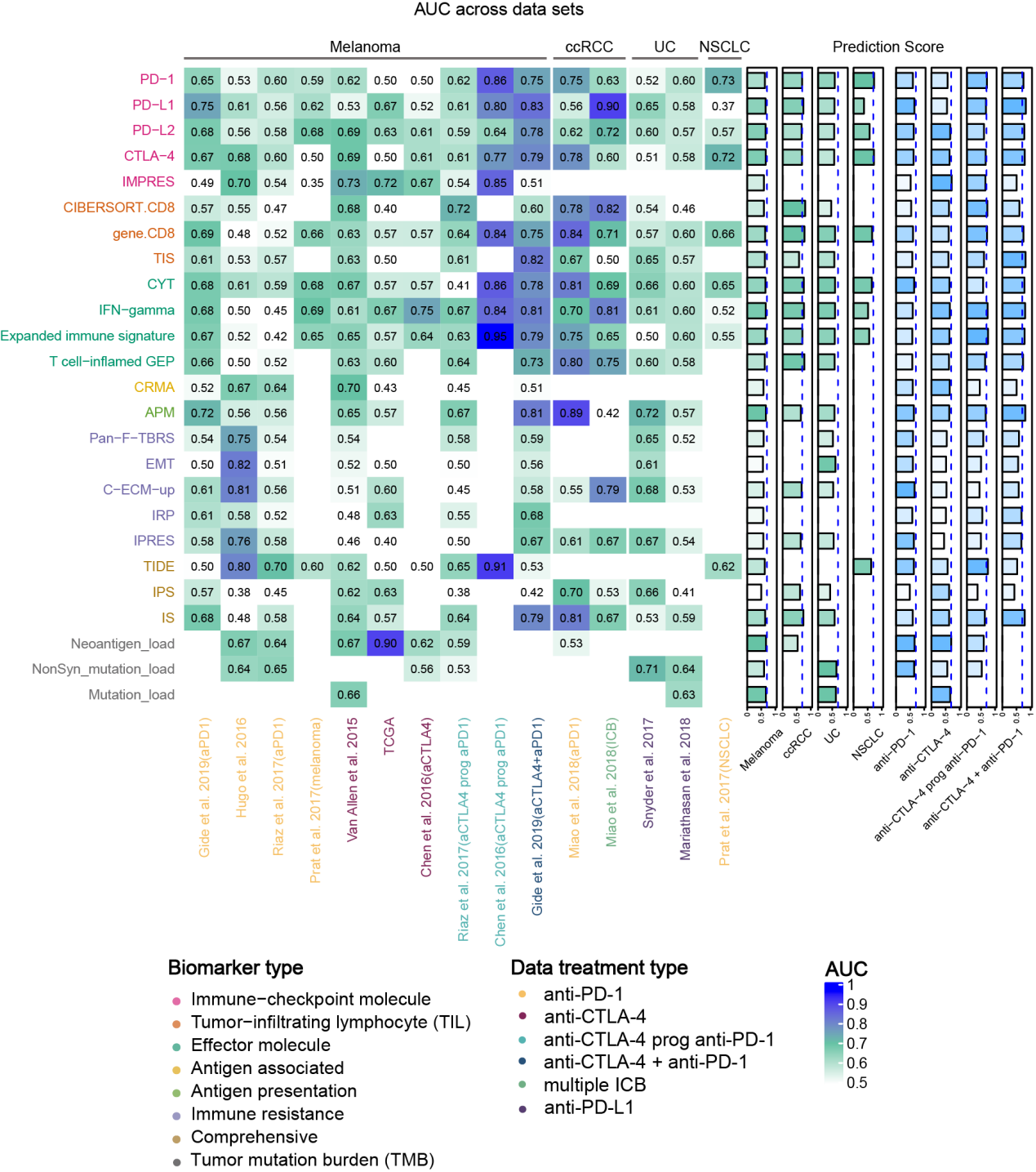


**Figure S21.** (related to Figure 5). Prediction performance of each biomarker for ICB response across different cancer types and different ICB therapy strategies in melanoma. AUC across 15 benchmark datasets and the prediction score (sum of sample size-weighted AUC) of different biomarkers were shown. Left panel: The color of heatmap indicated the prediction performance. Right panel: the color gradient and height of bar indicated the prediction score from low (light) to high (dark).


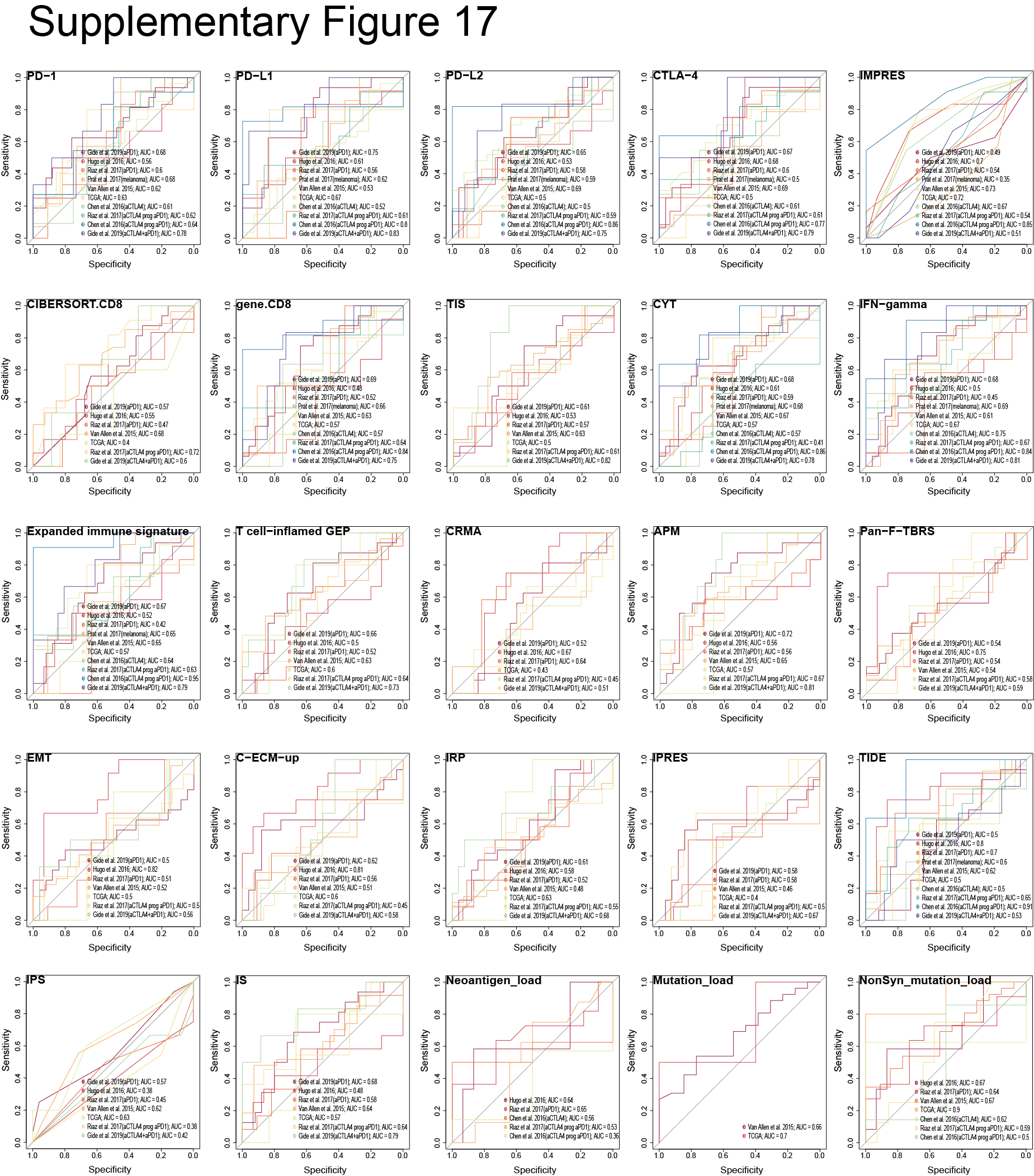


**Figure S22.** (related to Figure 5). Prediction performance of each biomarker for ICB response **across different ICB therapy strategies in melanoma.** ROC curves quantifying the prediction accuracy of different biomarkers in 10 melanoma datasets.


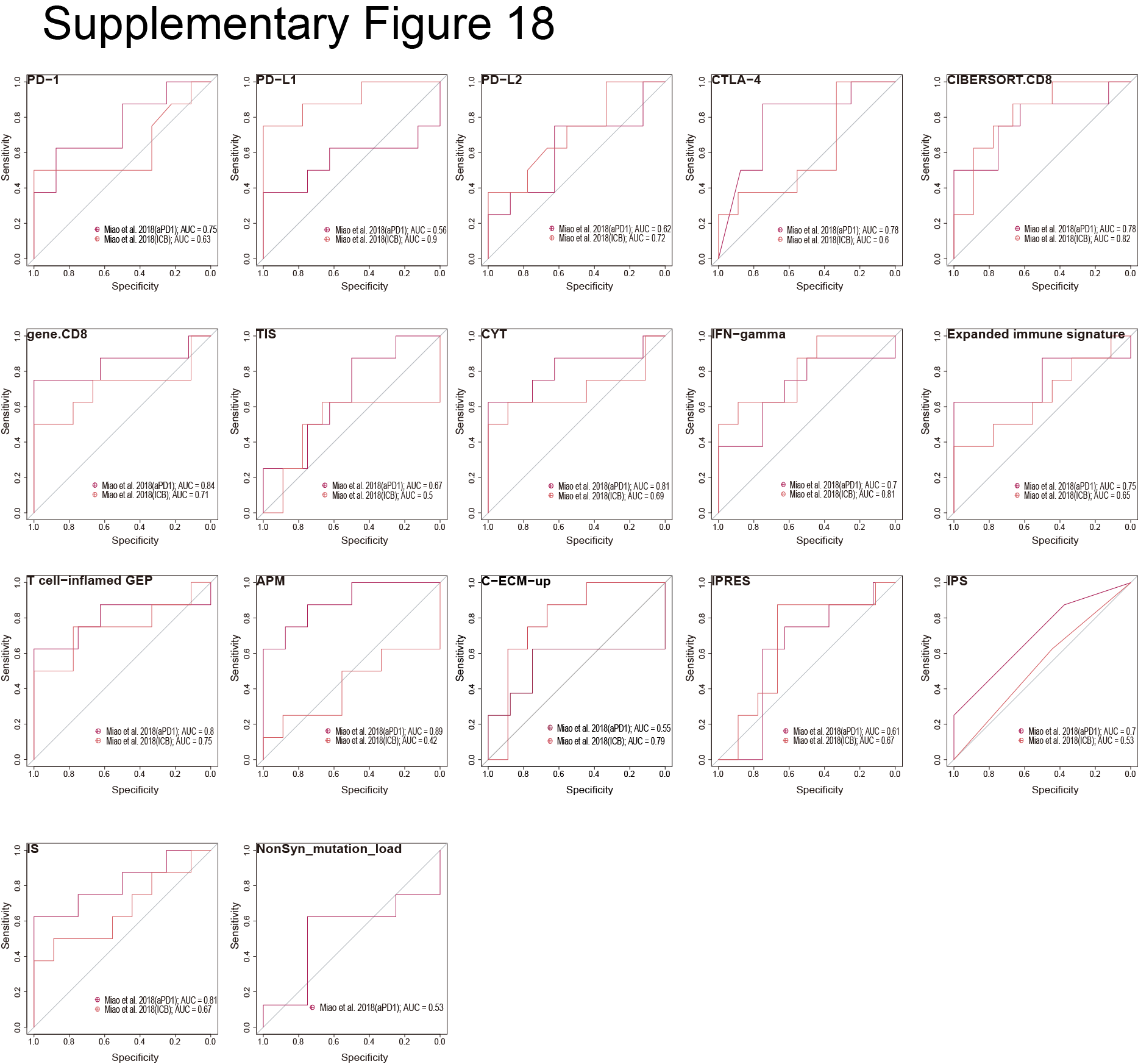


**Figure S23.** (related to Figure 5). Prediction performance of each biomarker for ICB response **in ccRCC.** ROC curves quantifying the prediction accuracy of different biomarkers in two ccRCC datasets.


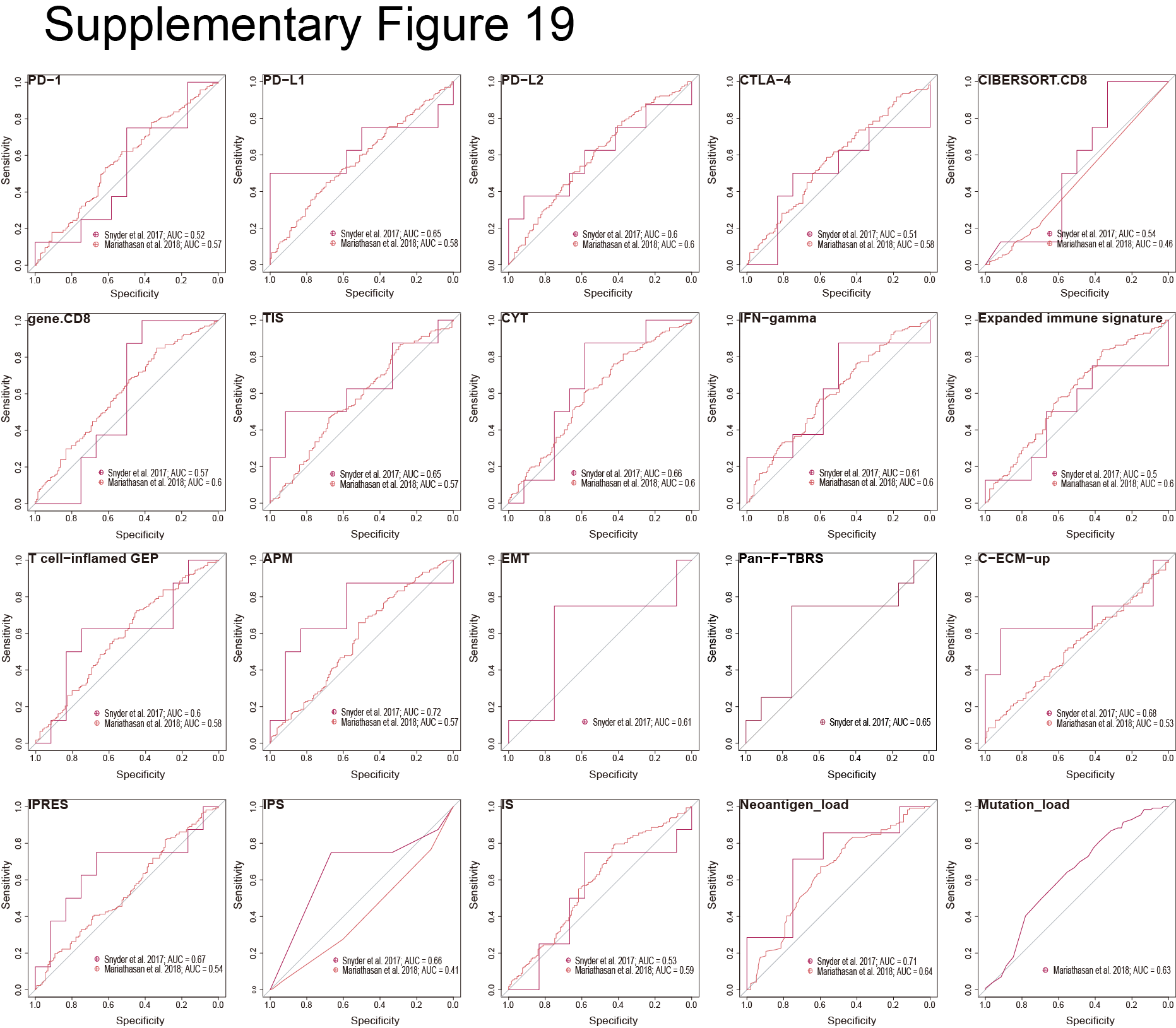


**Figure S24.** (related to Figure 5). Prediction performance of each biomarker for ICB response **in UC.** ROC curves quantifying the prediction accuracy of different biomarkers in two UC datasets.


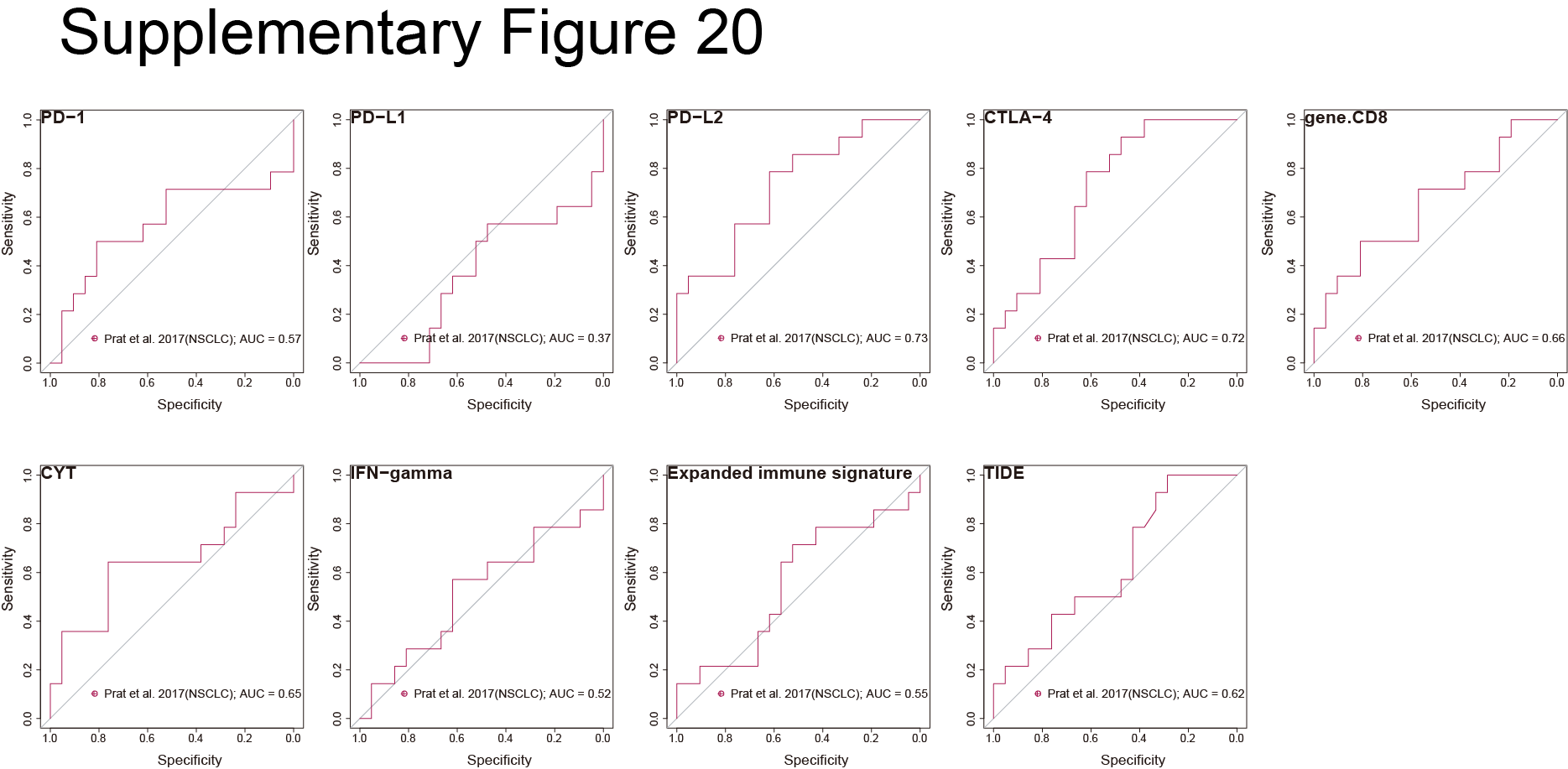


**Figure S25.** (related to Figure 5). Prediction performance of each biomarker for ICB response **in NSCLC.** ROC curves quantifying the prediction accuracy of different biomarkers in one NSCLC dataset.


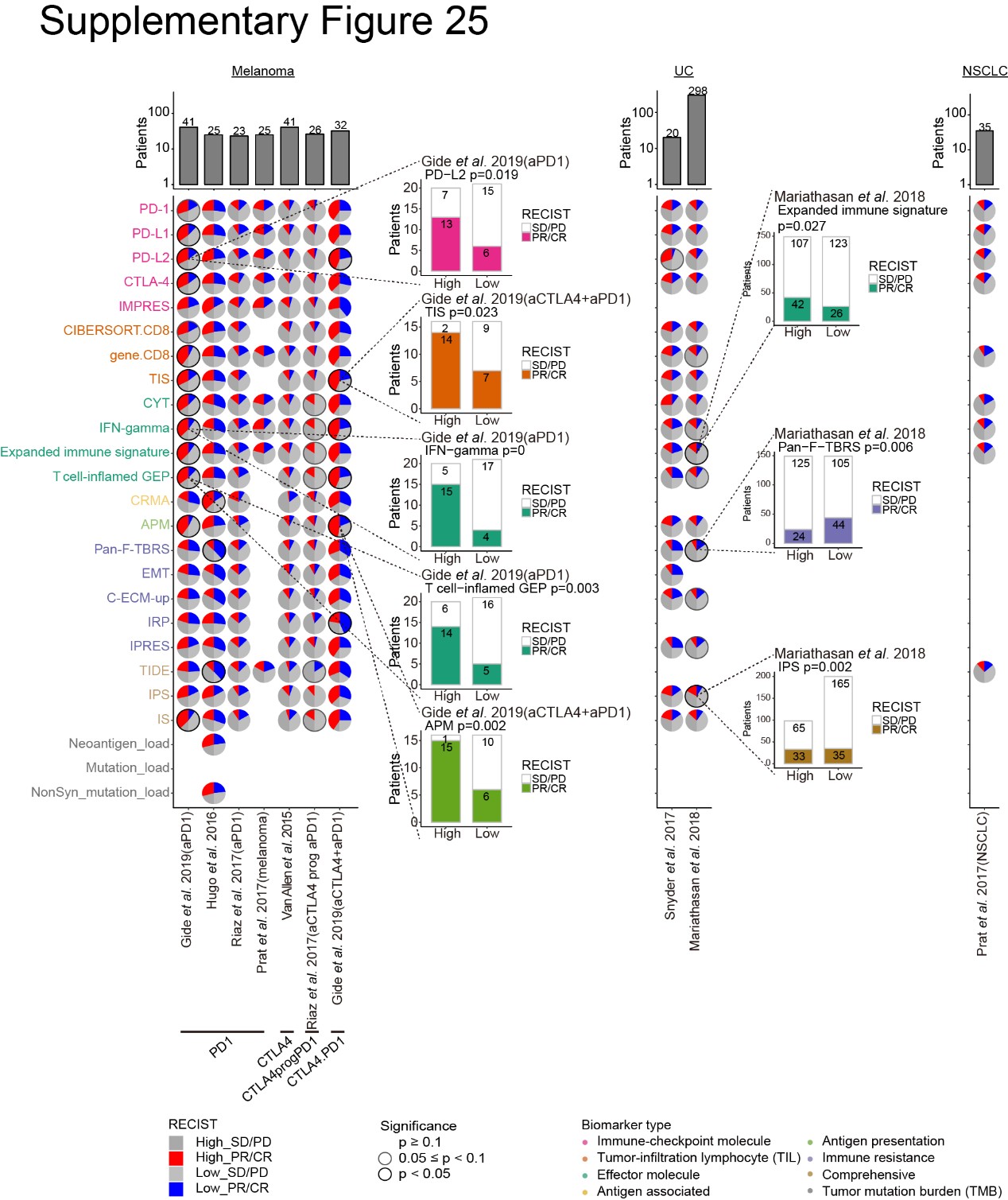


**Figure S26.** (related to Figure 6). Evaluation of the associations between the biomarkers and objective response to ICB across different cancer types and different ICB therapies**.** The significance of associations between biomarkers and objective response to ICB in each benchmark dataset across cancer types and ICB therapy strategies. Red and dark gray indicated the frequency of responders and non-responders in patients with high scores of the corresponding biomarkers, respectively. Blue and light gray indicated the frequency of responders and non-responders in patients with low scores of the corresponding biomarkers, respectively. Borders with no color, gray borders and black borders represented *p* ≥ 0.1, 0.05 ≤ *p* < 0.1, *p* < 0.05, respectively. Different categories of biomarkers were represented by different colors. Datasets with less than 20 samples were excluded from the analysis.
